# Multiple benefits of herbs: Polygonaceae species in veterinary pharmacology and livestock nutrition

**DOI:** 10.1016/j.vas.2024.100416

**Published:** 2024-12-02

**Authors:** Zafide Türk, Florian Leiber, Theresa Schlittenlacher, Matthias Hamburger, Michael Walkenhorst

**Affiliations:** a Research Institute of Organic Agriculture (FiBL), Ackerstrasse 113, 5070, Frick, Switzerland; b University of Basel, Department of Pharmaceutical Sciences, Klingelbergstrasse 50, 4056, Basel, Switzerland

**Keywords:** Buckwheat, Knotweed, Dock, Forage, Bioactive phytochemicals, Animal health, Animal nutrition

## Abstract

Herbs rich in secondary metabolites may possess beneficial properties in livestock nutrition and health. 49 Polygonaceae species of European mountain regions were included in a qualitative systematic review based on the methodological framework of the PRISMA statement. 174 relevant publications were identified. They comprised 231 *in vitro* and 163 *in vivo* experiments with cattle, sheep, goats, poultry, pigs, and rodents. For 16 Polygonaceae species no reports were found. *Fagopyrum esculentum* and *Fagopyrum tataricum* showed potential as anti-inflammatory, antioxidative and metabolic modifying herbs and feeds improving intake and nitrogen conversion in broiler as well as milk quality and ruminal biotransformation in dairy cows. *Polygonum aviculare* was promising as an antimicrobial and anti-inflammatory drug or feed, improving performance and affecting ruminal biotransformation in sheep, and *Polygonum bistorta* as an anti-inflammatory drug or feed, improving performance in broiler and mitigating methane emissions in ruminants. *Rumex obtusifolius* showed potential as an antibacterial drug or feed improving ruminal biotransformation and preventing bloating in cows, while *Rumex acetosa* and *Rumex acetosella* had antimicrobial and anti-inflammatory properties. Furthermore, *Polygonum minus, Polygonum persicaria, Rumex crispu*s and *Rumex patienti*a possess interesting anti-inflammatory and antimicrobial activities. In conclusion, some Polygonaceae species show relevant properties that might be useful to prevent and treat livestock diseases, combined with nutritional benefits in performance, product quality, lowering ruminal methane and ammonia formation and transferring omega-3 fatty-acids from feed to tissue. The potential of such multifunctional plants for a holistic integration of veterinary, nutritional and ecological perspectives under a one-health approach of livestock management is discussed.

## Highlights

• Beneficial properties of Polygonaceae in animal nutrition and health.
• Relevant information about 11 species of Polygonaceae was found.
• Potentiality of Polygonaceae in nutrition, disease prevention, and ecology.

## 1 Introduction

The Polygonaceae family includes about 1’200 species worldwide in approximately 50 genera (Lajter et al., 2013). Forty-seven species are part of Swiss native flora, which can be considered as representative for the European sub-alpine and alpine region. Some Polygonum and Rumex species frequently grow in grassland (*Polygonum alpinum* All., *Polygonum bistorta* L., *Polygonum viviparum* L., *Rumex acetosa* L., *Rumex alpestris* Jacq., *Rumex alpinus* L., *Rumex crispus* L., *Rumex longifolius* DC., *Rumex obtusifolius* L. and *Rumex thyrsiflorus* Fingerh.) (Flora, 2023). These species are foraged by ruminants or other livestock, either deliberately on pastures (Gorlier et al., 2012) or randomly in conserved feed. However, none of the Polygonaceae are established by purpose in agricultural grassland (UFA-Samen, 2024). Plant parts of wild or cultivated Polygonaceae species can be used as animal feed and human food (*e.g. Rumex acetosa* L., *Rheum rhabarbarum* L., and two *Fagopyrum* species providing grain (Christa & Soral-Smietana, 2008; Lajter et al., 2013; Leiber, 2016).

Furthermore, several Polygonaceae species are described in Swiss and European ethnoveterinary research and also in historical veterinary pharmacology textbooks for being used in European traditional veterinary medicine. For example, *Rumex obtusifolius* L. has a wide range of use in ethnoveterinary medicine, including treatment of diseases of the gastrointestinal tract and metabolism, skin, genitourinary system, udder, musculoskeletal system and respiratory system. (Disler et al., 2014; Stucki et al., 2019; Schlittenlacher et al., 2022). The family of Polygonaceae synthesize a broad variety of polyphenols and further bioactive compounds including flavonoids (*e.g.* rutin), gallotannins, naphthodianthones, anthraquinones (*e.g.* fagopyrin), stilbenoids and phenylpropanoids (Cherian et al., 2023; Huda et al., 2021), and various vitamins and minerals. In their natural composition, these plant secondary metabolites can have both pharmacological and nutritional effects. Concentration of such compounds is always highly depending on plant part (Leiber et al., 2012) and phenological stage (Kälber et al., 2014; Kreft et al., 2016). These compounds may have beneficial metabolic and health effects, in particular through antioxidative and antimicrobial properties (Nan et al., 2024; Zhang et al., 2015a). Furthermore, rutin bears effective protection of organs against various toxins (Rahmani et al., 2022).

There are numerous examples showing that bioactive plant compounds in livestock nutrition affect the nutritional quality of animal products (Leiber et al., 2005; Provenza et al., 2019; van Vliet et al., 2021). For instance, the level of natural antioxidants (such as rutin from buckwheat) in animal feed matters not only for animal health (Hassan et al., 2019), but also for increased vitamin E concentrations in livestock products (Leiber & Messikommer, 2011). Plant secondary metabolites in forage herbs modulate ruminal lipid metabolism by increasing the concentration of essential unsaturated fatty acids in ruminant products (Buccioni et al., 2012). Relevant to say, increased unsaturated fatty acids in animal tissues are first and foremost relevant for the physiology of the animal itself (Leiber et al., 2019). These examples imply that plant diversity and phytochemical richness in livestock feed has positive effects on animal welfare and health (Beck & Gregorini, 2020; Leiber et al., 2020b), as well as on human wellbeing via the increased nutritional quality of the products (Provenza et al., 2015). Furthermore, dietary herbs with relevant concentrations of secondary metabolites like tannins lower the ruminal production of methane (Hristov et al., 2022) and ammonia, and influence the nitrogen degradation (Molan et al., 2007) and efficiency in ruminants (Kapp-Bitter et al., 2023; Leiber et al., 2020a; Zhang et al., 2019). Feeding tannin-rich herbs as a potential anti-parasitic feed-additive has been shown to reduce methane and CO_2_ emissions in the mesocosm of the respective livestock farm (Verdú et at., 2020).

On the other hand, Polygonaceae also possess antinutritive properties as for example phytic acid and trypsin inhibitors, which may significantly inhibit mineral absorption, release and activity of trypsin, and cause tissue damages in the digestive tract (Berrens and Maranon, 1995; Nan et al., 2024; Zhang et al., 2015b). Protease inhibitors, however, also have antimicrobial, antifungal and antitumor effects (Chen & Ruan, 2016). Furthermore, the phototoxic fagopyrin from buckwheat and polygonum species may evoke photodermatitis and digestive disorders, which are scarcely described in scientific literature, though (Benkovic et al., 2014; Leiber, 2016). Concentration and severity of antinutritive factors and phototoxins also largely depend on plant part and developmental phase (Nan et al., 2024; Ozbolt et al., 2008; Zhang et al., 2015b).

In conclusion, there is obviously considerable overlap among nutritive, toxicological, and pharmaceutical properties of Polygonaceae species with respect to animals. This makes them interesting for a One-Health perspective on natural feed production, feeding practices and veterinary concepts regarding livestock. The One Health concept focuses on the transmission chains for pathogens, in particular zoonotic diseases (Sinclair, 2019; WHO, 2023), acknowledging that, besides sanitary and hygienic means, the general health of ecosystems, production systems and social communities have to be addressed in an interdisciplinary and holistic approach. In a perspective of preventive measures that foster human and animal wellbeing, resilience and robustness, a healthy and diverse nutrition plays a major role (Leiber et al., 2020b). With respect to Polygonaceae, there is evidence that they possess several of the above-mentioned nutritional (Islam et al., 2016; Leiber, 2016) and veterinary (Ayrle et al., 2016) properties. Thus, an analysis of parallel existing evidence for veterinary, nutritional, and environmental effects, narrowed down to this one botanical family, could provide an example for a holistic synopsis of nutritional and health aspects for livestock and humans in the agri-food chain.

Against this background, the aim of this systematic review was to compile, based on recent *in vitro, in vivo* and clinical research, an overview on how European alpine and sub-alpine native, introduced, or cultivated Polygonaceae species might be promising candidates (a) to increase feed diversity and richness in secondary metabolites, (b) improve livestock performance and quality of livestock products, and (c) to prevent and/or treat livestock diseases.

To the authors knowledge we conducted for the first time a review covering both the animal health and nutritional properties of plant species of the Polygonaceae family.

## 2 Material and methods

The recommendation of the PRISMA statement (Liberati et al., 2009) and the AMSTAR measurement tool (Shea et al., 2007) served as the basis for the design of the systematic review. The research question was designed following the PICOS scheme (Liberati et al., 2009):

Population were ruminants and other livestock species plus rodents, and the intervention was a treatment with raw material or extracts based on European alpine and sub-alpine Polygonaceae species. The comparator was either no treatment, a placebo, or a standard treatment. The outcome was the effect of the plant material or the extract of Polygonaceae species. The study design included *in vitro* (including rumen model), *in vivo* and clinical data (Additional file 1).

### 2.1 Relevance screening and selection of Polygonaceae species

To choose eligible Polygonaceae species, different initial sources were screened with regard to species frequently recommended for the treatment of livestock, and for their occurrence in the Swiss flora (Flora, 2023). The flora of Switzerland can be considered as representative for the European alpine Polygonaceae, given that it covers northern and southern alpine and sub-alpine slopes and all altitudes. As initial sources three historic veterinary pharmacology textbooks (Delafond, 1853; Fröhner, 1900; Röll, 1866), two publications focusing on European ethnoveterinary plant use (Mayer et al., 2014; Vogl et al., 2016), two publications on alpine forage plant species (Gorlier et al., 2012; Jayanegara et al., 2011), and a list of all plant species reported in recent ethnoveterinary studies in Switzerland (Bischoff et al., 2016; Disler et al., 2014; Mayer et al., 2017; Mertenat et al., 2020; Schmid et al., 2012; Stucki et al., 2019) were screened in order to identify relevant Polygonaceae species. Out of these seven sources the following information about all species of the Polygonaceae family were extracted: plant species, plant part, ATC vet Code and type of administration. All further 32 Swiss indigenous Polygonaceae species (including the 3 neophytic species of the Genus *Reynoutria*; Flora, 2023) were included in addition (Table 1).

**Table 1:** Selection of relevant Polygonaceae species.

Plant species	Initial sources		ATC vet Code^1^	EEV countries^2^	Vet. Pharm. textbook^3^
	EEV	SNF			
*Fagopyrum esculentum* Moench	x	x	QG (2)	A, CH	
*Fagopyrum tataricum* (L.) Gaertn.		x			
*Fallopia aubertii* (L. Henry) Holub		x			
*Fallopia convolvulus* (L.) A. Löve		x			
*Fallopia dumetorum* (L.) Holub		x			
*Oxyria digyna* (L.) Hill		x			
*Polygonum alpinum* All.		x			
*Polygonum amphibium* L.		x			
*Polygonum arenastrum* Boreau		x			
*Polygonum aviculare* L.	x	x	QA (1), QD (2), n.a (1) GS (1)	CH, G, E, I	
*Polygonum bistorta* L.	x	x	QA (4), n.a (2)	I	x (Delafond, 1853; Röll, 1866)
*Polygonum capitatum* D.Don		x			
*Polygonum hydropiper* L.		x			
*Polygonum lapathifolium* L.		x			
*Polygonum minus* Huds.		x			
*Polygonum mite* Schrank		x			
*Polygonum nepalense* Meisn.		x			
*Polygonum orientale* L.		x			
*Polygonum perfoliatum* L.		x			
*Polygonum persicaria* L.	x	x	QG (1)	E	
*Polygonum polystachyum* Meisn.		x			
*Polygonum rurivagum* Boreau		x			
*Polygonum viviparum* L.		x			
*Reynoutria x bohemica* Chrtek & Chrtkova		x			
*Reynoutria japonica* Houtt.		x			
*Reynoutria sachalinensis* (F. Schmidt) Nakai		x			
*Rheum officinale* Baill./*palmatum* L.	x		QR (1), QA (6)	HR	x (Fröhner, 1900)
*Rheum rhabarbarum* (L.)	x	x	QA (5), QV (1)		x (Röll, 1866)
*Rumex x pratensis* Mert. & W.W.J. Koch		x			
*Rumex acetosa* L.	x	x	Feed/food integrator (1), QA (1), QP (3), QR (1), others (3)	I, E	x (Delafond, 1853)
*Rumex acetosella* L.	x	x	QA (1)	HR	
*Rumex alpestris* Jacq.	x	x			
*Rumex alpinus* L.	x	x	Feed/food integrator (3), QA (2), QD (6), QG52 (1), QM (1)	AL, I, CH	
*Rumex aquaticus* L.		x			
*Rumex conglomeratus* Murray	x	x	QA (1)	CH	
*Rumex crispus* L.	x	x	Feed/food integrator (1), QA (3), QD (1)	I	
*Rumex hydrolapathum* Huds.		x			
*Rumex longifolius* DC.		x			
*Rumex maritimus* L.		x			
*Rumex nebroides* Campd.		x			
*Rumex nivalis* Hegetschw.		x			
*Rumex obtusifolius* L.	x	x	Feed/food integrator (1), QA (16), QD (25), QG (1), QG52 (10), QM (14), QR (1), QV (4)	G, I, CH	x (Röll, 1866)
*Rumex palustris* Sm.		x			
*Rumex patientia* L.		x			
*Rumex pulcher* L.	x	x	Feed/food integrator (2), QP (1)	E	
*Rumex sanguineus* L.	x	x	Feed/food integrator (1)	I	
*Rumex scutatus* L.		x			
*Rumex* sp.	x		Feed/food integrator (3), QA (4), QG (1)	SRB, RO, I	
*Rumex thyrsiflorus* Fingerh.		x			

EEV: European ethnoveterinary research, SNF: Swiss native flora including 3 species of the neophytic genus *Reynoutria.*

1 ATC vet Code in European ethnoveterinary research and/or historical textbooks of veterinary pharmacology, QA: Alimentary tract and metabolism, QD: Dermatological, QG: Genitourinary system and sex hormones, QG52: Mastitis, QP: Antiparasitic products, insecticides and repellents, QR: Respiratory system, QV: Various, GS: General strengthening.

2 ethnoveterinary use in European countries, A: Austria (Vogl et al., 2016), CH: Switzerland (Bischoff et al., 2016; Disler et al., 2014; Mayer et al., 2017; Mertenat et al., 2020; Stucki et al., 2019; Schmid et al., 2012) , E: Spain (Bonet et al., 2007; Gonzalez et al., 2011), G: Germany (Bavaria; Schlittenlacher et al., 2022), I: Italy )Viegi et al., 2003; Manganelli et al. 2001), HR: Croatia (Pieroni et al., 2003; Vucevac-Bajt et al., 1994), AL: Albania (Pieroni et al., 2005), SRB: Serbia (Jari et al., 2007), RO: Romania (Papp et al., 2012)

3 historical textbooks of veterinary pharmacology (Delafond, 1853; Fröhner, 1900; Röll, 1866)

### 2.2 Selection of scientific references

#### 2.2.1 Bibliographic search

Scientific information on these Polygonaceae species were searched in PubMed (PubMed.gov, 2015) and Web of Science (Web of Science, 2020). Both bibliographic sources were consulted in the time between 20.01.2021 and 27.01.2021 by one person. First, we created a search term which consisted of the Latin name, the English name and, if applicable, the pharmaceutical herbal drug name of the respective Polygonaceae species. We excluded information on use or effects in neoplastic diseases. This led to the following search term: (“Latin name” OR “English name” OR “herbal drug”) NOT (cancer* OR anticancer* OR anti-cancer* OR anti-tumor* OR antitumor* OR tumor*). The search term was used for each Polygonaceae species separately. The second part of the English name of some Polygonaceae species were knotweed, knotgrass and sorrel. Therefore, the search for these names was conducted in addition with the search term: (“knotweed” OR “knotgrass” OR “sorrel”) NOT (cancer* OR anticancer* OR anti-cancer* OR anti-tumor* OR antitumor* OR tumor*).

The year of publication was not narrowed down. In Web of Science searches the results were refined with the following research areas: Agricultural, Agriculture dairy animal science, Agriculture multidisciplinary, Agronomy, Allergy, Behavioural science, Biochemical research methods, Biochemistry molecular biology, Biology, Biotechnology applied microbiology, Cell biology, Chemistry analytical, Chemistry applied, Chemistry medicinal, Chemistry multidisciplinary, Chemistry organic, Chemistry physical, Dermatology, Engineering, Engineering chemical, Entomology, Food science technology, Green sustainable science technology, Immunology, Integrative complementary medicine, Microbiology, Multidisciplinary science, Mycology, Nutrition dietetics, Pharmacology and pharmacy, Physiology, Substance abuse, Surgery, Toxicology, Veterinary science, Virology, Zoology.

All detected publications were saved in one folder per species in an EndNote® 20 database.

#### 2.2.2 Removing duplicates automatically and manually

The publications where the species could be clearly identified, were manually assigned from the folders knotweed, knotgrass and sorrel to the corresponding folders of Polygonaceae species. After that, duplicates were removed within the folder of each species. Duplicates which were not automatically detected were removed manually afterwards.

#### 2.2.3 Refining with manual title and abstract check

Only peer-reviewed publications with an abstract written in English, German, Turkish, French or Spanish were considered for further evaluation. The manual title and abstract check was conducted separately for each Polygonaceae species by one person based on predefined detailed in- and exclusion criteria (chapter 2.2.4; Additional file 1). Only publications reporting *in vivo* and *in vitro* experiments were included. If the title or abstract was not sufficiently informative for decision making, the full text was also consulted (Additional file 1).

#### 2.2.4 Definition of inclusion and exclusion criteria

The inclusion and exclusion criteria were pre-defined by three scientists. Included were publications that clearly contained a control group (placebo, untreated or positive control) , one of the Polygonaceae species and *in vitro* (including rumen model), *ex vivo*, or *in vivo*, nutritional, clinical or phytochemical data. A publication was excluded if it dealt with other plant species or with mixtures of different plant species, food quality, technology and packaging, plant genetics, cultivation or breeding of plants, weed control, plant pathology, fertilizers, plant protection systems or pesticides, ecology, geology, ethology, sociology, and ethnobotany (Additional file 1).

### 2.3 Definition and categorization of the publications (including one or more experiments)

After abstract check the publications were categorized into *in vivo, in vitro*, food processing, pharmacognosy, side effects/toxicology, various information and reviews (Table 2). For further assessment, the focus was only on *in vivo* and *in vitro* studies.

**Table 2:** Details on the process of quantifying and categorizing scientific publications during the bibliographic search process.

Basic information				Further information					Included information (Main issue of the publication)							
Plant species	Remaining publications			Review	Side effect/ Toxicology	Various information	Pharma-cognostic	Food processing	*In vivo*			*In vitro*				Total publications
	after removal of duplicates	after title check	after abstract/ full text check						Livestock	Laboratory animals	Human	Livestock^1^	Laboratory animals^2^	Human^3^	Others^4^	
*Fagopyrum esculentum* Moench	4122	392	281	35	41	13	58	158	21	24	8	4	0	8	0	**65**
*Fagopyrum tataricum* (L.) Gaertn.	342	63	48	2	2	9	13	17	1	12	2	1	1	1	0	**18**
*Fallopia aubertii* (L. Henry) Holub	2	2	1	0	1	0	1	0	0	0	0	0	0	0	0	**0**
*Fallopia convolvulus* (L.) A. Löve	365	5	3	0	2	0	2	0	0	1	0	0	0	0	0	**1**
*Fallopia dumetorum* (L.) Holub	311	0	0	0	0	0	0	0	0	0	0	0	0	0	0	**0**
*Oxyria digyna* (L.) Hill	25	1	1	0	0	0	1	0	0	0	0	0	0	0	0	**0**
*Polygonum alpinum* All.	4	1	1	0	0	0	1	0	0	0	0	0	0	0	0	**0**
*Polygonum amphibium* L.	36	2	2	0	0	0	0	0	0	0	0	0	0	0	2	**2**
*Polygonum arenastrum* Boreau	7	1	1	0	0	0	0	0	0	0	0	0	0	0	1	**1**
*Polygonum aviculare* L.	216	37	31	0	0	1	13	2	1	4	1	3	0	2	6	**17**
*Polygonum bistorta* L.	154	26	20	0	0	2	11	0	1	2	0	2	2	1	2	**10**
*Polygonum capitatum* D.Don	37	8	6	0	1	1	5	0	0	1	0	0	0	0	0	**1**
*Polygonum hydropiper* L.	171	43	28	0	9	3	16	1	0	2	0	2	2	0	5	**11**
*Polygonum lapathifolium* L.	47	4	4	0	0	0	2	0	0	1	0	0	0	0	1	**2**
*Polygonum minus* Huds.	172	21	18	0	1	1	9	3	0	3	0	0	0	2	1	**6**
*Polygonum mite* Schrank	3	3	3	0	0	0	2	0	0	0	0	0	0	0	1	**1**
*Polygonum nepalense* Meisn.	3	0	0	0	0	0	0	0	0	0	0	0	0	0	0	**0**
*Polygonum orientale* L.	64	8	7	0	0	1	6	0	0	1	0	0	0	0	0	**1**
*Polygonum perfoliatum* L.	61	9	7	0	0	2	5	0	0	1	0	0	0	0	1	**2**
*Polygonum persicaria* L.	88	6	5	0	0	0	2	0	0	0	0	0	0	0	3	**3**
*Polygonum polystachyum* Meisn.	7	2	2	0	0	0	2	0	0	0	0	0	0	0	0	**0**
*Polygonum rurivagum* Boreau	1485	4	1	0	3	0	1	0	0	0	0	0	0	0	0	**0**
*Polygonum viviparum* L.	40	3	1	0	0	1	1	0	0	0	0	0	0	0	0	**0**
*Reynoutria x bohemica* Chrtek & Chrtkova	11	2	2	0	0	0	1	0	0	0	0	0	0	1	0	**1**
*Reynoutria japonica* Houtt.	151	17	11	0	0	3	8	1	0	0	0	0	0	1	0	**1**
*Reynoutria sachalinensis* (F. Schmidt) Nakai	75	9	4	0	2	3	3	0	0	0	0	0	0	1	0	**1**
*Rheum officinale* Baill./*palmatum* L.	619	69	27	3	4	16	9	1	1	4	1	3	3	1	4	**17**
*Rheum rhabarbarum* (L.)	45	19	12	1	1	3	7	4	0	0	0	0	0	0	1	**1**
*Rumex x pratensis* Mert. & W.W.J. Koch	10	0	0	0	0	0	0	0	0	0	0	0	0	0	0	**0**
*Rumex acetosa* L.	193	18	16	0	1	0	3	6	1	2	0	0	1	2	1	**7**
*Rumex acetosella* L.	98	12	10	0	2	0	3	4	0	1	0	0	0	0	2	**3**
*Rumex alpestris* Jacq.	0	0	0	0	0	0	0	0	0	0	0	0	0	0	0	**0**
*Rumex alpinus* L.	17	2	2	0	0	0	0	0	0	0	0	0	0	0	2	**2**
*Rumex aquaticus* L.	17	13	1	0	0	12	0	0	0	0	0	0	0	0	1	**1**
*Rumex conglomeratus* Murray	113	2	1	0	1	0	0	0	0	0	0	0	0	0	1	**1**
*Rumex crispus* L.	187	24	16	0	4	2	5	4	0	1	0	0	1	1	4	**7**
*Rumex hydrolapathum* Huds.	13	1	1	0	0	0	0	0	0	0	0	0	0	0	1	**1**
*Rumex longifolius* DC.	4	1	0	0	0	0	0	0	0	0	0	0	0	0	0	**0**
*Rumex maritimus* L.	11	2	2	0	0	0	1	0	0	1	0	0	0	0	0	**1**
*Rumex nebroides* Campd.	719	0	0	0	0	0	0	0	0	0	0	0	0	0	0	**0**
*Rumex nivalis* Hegetschw.	8	0	0	0	0	0	0	0	0	0	0	0	0	0	0	**0**
*Rumex obtusifolius* L.	220	24	21	0	0	4	4	7	3	1	0	1	0	0	4	**9**
*Rumex palustris* Sm.	92	0	0	0	0	0	0	0	0	0	0	0	0	0	0	**0**
*Rumex patientia* L.	58	16	11	0	1	0	4	1	0	3	0	0	0	0	3	**6**
*Rumex pulcher* L.	13	6	6	0	0	0	1	4	0	0	0	0	0	0	1	**1**
*Rumex sanguineus* L.	4	0	0	0	0	0	0	0	0	0	0	0	0	0	0	**0**
*Rumex scutatus* L.	10	4	4	0	0	0	1	2	0	0	0	0	0	0	1	**1**
*Rumex* sp.	164	14	13	0	1	0	5	8	0	0	0	0	0	0	0	**0**
*Rumex thyrsiflorus* Fingerh.	9	1	1	0	0	0	0	0	0	0	0	0	0	0	1	**1**

1 Rumen model, cell lines.

2/3 Cell lines.

4 Bacterial, viral, fungal cultures.

Trials investigating effects of biomass or preparations from Polygonaceae species on diseases, digestion, performance, or other parameters in living animals and humans were categorized as “*in vivo*”. Investigations using pathogens, cellular or *ex vivo* models (*e.g.* rumen fermentation models) were categorized as “*in vitro*”. Subgroups were formed for *in vivo* (including (a) livestock, (b) laboratory animals and (c) human) and *in vitro* (including (a) livestock directed research like rumen model or livestock-derived cell lines, (b) laboratory animal-derived cell lines, (c) human cell lines, and (d) further research mainly utlizing bacterial, fungal or viral cultures).

The finally included publications were categorized by one person into two main groups: “*in vivo*” and “*in vitro*” publications. *In vivo* publications were papers with *in vivo* data even if they included in addition *in vitro* data, while *in vitro* publications contained solely *in vitro* data. Given that publications often reported testing of several extracts of a given plant and, also both *in vivo* and *in vitro* data, we defined the “experiment” as key “unit” in our review, subdivided into *in vivo* and *in vitro* experiments.

*In vivo* experiments: Each trial within different or the same publication documenting two or more groups of animals kept under the same conditions during the same time period, receiving raw material or an extract of one plant part (or of the whole plant) of a Polygonaceae species (publication x trial x plant species x plant part x extracting agent) compared to a control group.

Thus, a publication including two trials with *Fagopyrum esculentum* Moench, one in turkeys and one in chicken was counted as 2 experiments. A publication reporting a trial with 10 groups of animals receiving different plant species, two of them from the Polygonaceae family (*e.g. Fagopyrum esculentum* Moench or *Fagopyrum tataricum* (L.) Gaertn.) was also counted as 2 experiments. A publication reporting a trial with three different extracts of *Rumex maritimus* L. was counted as 3 experiments (three lines in Additional file 2).

*In vitro* experiment: Each *in vitro* experiment in a publication in which a specific extract of a particular plant part (or a whole plant) of a plant species was tested for one or more effects (publication x plant species x plant part x extracting agent). Testing of an acqueous extract of *Rumex crispus* L. for antibacterial and antifungal effects by agar dilution was counted as 1 experiment (one line in Additional file 3), while the testing of four different extracts of *Polygonum aviculare* L., was counted as 4 experiments (four lines in Additional file 3).

### 2.4 Assessment of the experiments

Information were systematically recorded from *in vivo* and *in vitro* experiments. For *in vivo* experiments information about Polygonaceae species, plant part, extracting agent, animal species, total number of animals, control groups, experimental groups, topic, effects and physiological effect on the rumen were assessed (Additional file 2). For *in vitro* experiments the information on Polygonaceae species, plant part, extracting agent, topic (including type of method), effects and physiological effect on rumen were assessed (Additional file 3). For each investigated effect the outcomes were categorized as follows: Positive (p) meaning there was a significant positive effect; (no) meaning that no significant effect was observed; negative (n) meaning that significant negative effect was seen; question mark (?) meaning that the effect was inconsistent (Table 3). For example in *in vivo* experiments regarding performance a statistical significant higher daily weight gain, compared to an untreated control group, was counted as (p), while a lower daily weight gain was counted as (n) and no statistical difference as (no).

**Table 3:** Scoring system used in the systematic literature research for each parameter measured in each experiment.

Effects*		Experiments that compared a medicinal plant-based treatment only with an antiparasitic, antibacterial or another treatment as control.	Experiments that compared a medicinal plant-based treatment at least with a negative control group (placebo-treatment or no treatment), sometimes, in addition, with an antiparasitic, antibacterial or another treatment as control.
**p**	Positive effect (in case of several dosages of one plant material at least one dosage showed a positive effect, other dosages showed no effect).	The medicinal plant-based treatment showed a significant positive effect compared to any control group or no difference compared to the antiparasitic / antibacterial treatment	Medicinal plant-based treatment showed a **significant positive effect** compared to the negative control.
**no**	No significant effect compared to the negative control group	There is no significant difference compared to the negative control group or a significant negative difference compared to the antiparasitic / antibacterial treatment control	Medicinal plant-based treatment showed **no significant difference** to the negative control.
**n**	Negative effect (in case of several dosages of one plant material at least one dosage was negative, other dosages showed no effect)		Medicinal plant-based treatment showed a **significant negative effect** compared to the negative control.
**na**	No data available		
**?**	In case of experiments with several dosages of one plant material: if at least one dosage showed a positive and another dosage a negative effect compared to negative control group (inconsistent effect).		

⁎ as this study was not designed as a meta-analysis but more as a qualitative systematic review a detailed prove of the statistical methods was not conducted. Only the results that the authors presented as statistical significant were considered.

## 3 Results and discussion

### 3.1 Procedure of this systematic literature review

**Fig. 1:**
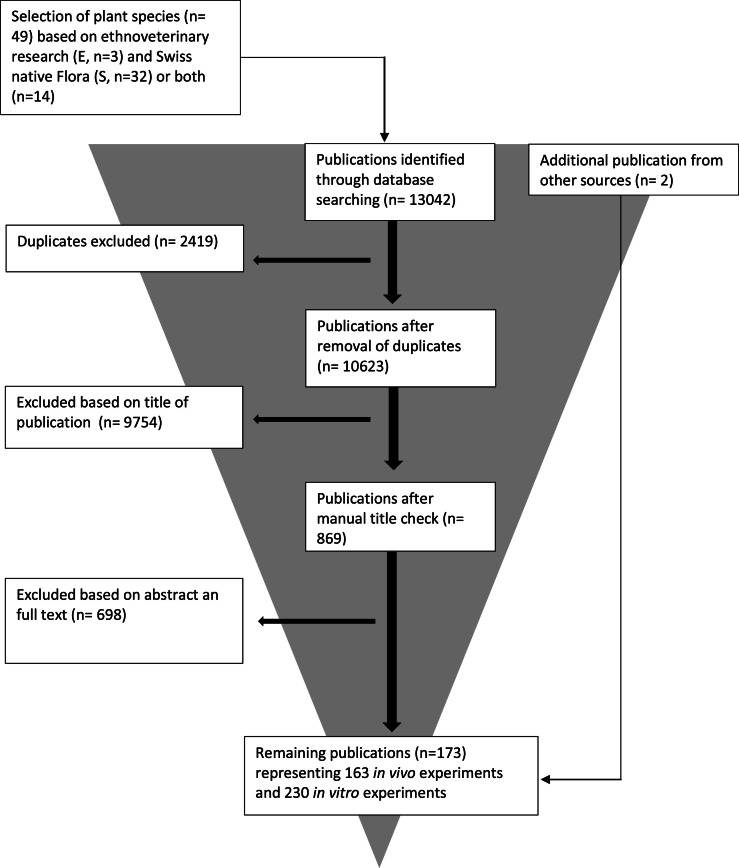
Selection process for publications included in the literature analysis.

The screening of ethnoveterinary research and Swiss native flora (initial sources) led to a total of 49 Polygonaceae species (Table 2). In the subsequent bibliographic search, 10’623 publications (after removal of duplicates) were retrieved. The following manual title and abstract check reduced the number to 869 potentially interesting publications. The detailed screening of these publications finally led to 173 publications (Fig. 1) representing 393 experiments (163 *in vivo* and 230 *in vitro*). A total of 10’450 publications were excluded because they did not match the predefined criteria. The main reason for exclusion was that the title and content of the abstract did not match with the focus of the review. Other reasons were lack of an abstract, or unavailability of publications online as full text. As our systematic review was not designed as a meta-analysis but more as a qualitative review a detailed prove of the statistical methods was not conducted. This might be a certain weakness. However, we only included studies with defined and clearly described control groups that conducted and provided a statistic.

#### 3.1.1 Frequencies of published evidence for Polygonaceae species

We found a large amount of evidence-based information on Polygonaceae, represented by 10’623 publications focusing on the selected 49 Polygonaceae species. However, in comparison with 30 plant species that are well known for their therapeutic use (Ayrle et al., 2016), we found on average about 3 times less publications per Polygonaceae species reporting experimental based knowledge about veterinary or nutritional use in farm animals.

In our review approximately four fifths of the publications were dealing with food processing, pharmacognosy, side effects/toxicology, or other data not of within the scope of this review, or they were reviews by themselves. Information from publications dealing solely with side effects and toxicology (75 mainly about *Fagopyrum esculentum* Moench) were taken into account in the discussion of the respective Polygonaceae species. For some species a high number of publications was identified in the initial search (more than 113 publications per species), but only approx. 10 % (*e.g.* for *Fagopyrum esculentum* Moench, *Fallopia convolvulus* (L.) A. Löve, *Polygonum minus* Huds., *Polygonum rurivagum* Boreau, *Reynoutria japonica* Houtt., *Rheum officinale/palmatum* L., *Rumex acetosa* L., *Rumex conglomeratus* MURRAY, *Rumex crispus* L., *Rumex obtusifolius* L.) or even none (*e.g.* for *Fallopia dumetorum* (L.) Holub, *Rumex nebroide*s Campd.) of these publications remained after title and/or abstract check.

More than 10 publications with *in vivo* or *in vitro* data were found for 7 Polygonaceae species, namely *Fagopyrum esculentum* Moench (65), *Fagopyrum tataricum* (L.) Gaertn. (18), *Polygonum aviculare* L. (17), *Polygonum bistorta* L. (10), *Polygonum hydropiper* L. (11), and *Rheum officinale/palmatum* L. (17). Less than 10 publications reporting *in vivo* or *in vitro* data were found for 26 Polygonaceae species, and for 16 species no publications with *in vivo* or *in vitro* data were identified. The number of publications containing *in vivo* information was comparable to that of papers describing only *in vitro* data.

### 3.2 Frequencies of published evidence

The 173 publications referred to 163 *in vivo* experiments (Additional file 2) and 230 *in vitro* experiments (Additional file 3).

#### 3.2.1 Veterinary / medical effects

Antibacterial effects of Polygonaceae species were investigated most frequently (148 times), followed by anti-inflammatory (75 times), antifungal (66 times), antihyperlipidemic (35 times) and antioxidant (32 times) properties (Table 4). Hepatoprotective (12 times), antiviral (10 times), antinociceptive/analgesic (10 times), antidiarrheal (9 times) and prebiotic (5 times) effects were less frequently reported for the 51 Polygonaceae species analyzed in this review.

**Table 4:** *In vivo* and *in vitro* effects reported for Polygonaceae species.

Plant species	Plant parts	Number of publications	Type of experiments	Antioxidative	Antiviral	Antibacterial	Prebiotic	Antifungal	Hepatoprotective	Anti-inflammatory	Antinociceptive/ analgesic	Antidiarrheal	Antihyperlipidemic		Livestock related effects	Other effects	Total effects of individual species x plant part			
				p/no/n	p/no/n	p/no/n	p/no/n	p/no/n	p/no/n	p/no/n	p/no/n	p/no/n	p/no/n		p/no/n/?		pos	none	neg	?
*Fagopyrum esculentum* Moench	Whole plant	1^1^	*In vitro*												1/1/-/-		**1**	**1**		
		4^2^	Ruminant												3/7/-/-		**3**	**7**		
		1^3^	LA												-/2/-/-			**2**		
	Grain/Seed	7^4^	*In vitro*	1/-/-									1/-/-		2/2/-/-	A	**4**	**2**	**1**	
		1^5^	Ruminant												-/2/-/-			**2**		
		8^6^	LA	2/2/-			1/-/-			-/1/-			6/2/-		4/13//2/-		**13**	**18**	**2**	
		6^7^	OA										1/-/-		5/5/1/-	B	**6**	**5**	**1**	
	Hull/Bran	1^8^	*In vitro*	1/-/-													**1**			
		3^9^	LA	1/-/-									1/-/-		-/3/-/-		**2**	**3**		
		4^10^	OA	1/-/-		1/1/-	-/2/-						-/1/-		3/5/-/-		**5**	**9**		
	n.a	4^11^	*In vitro*				1/-/-						1/-/-			C	**2**			
		3^12^	Ruminant												2/2/-/-		**2**	**2**		
		7^13^	LA							1/-/-			4/1/-		-/9/3/-		**5**	**10**	**3**	
		9^14^	OA	1/-/-						1/2/-			1/3/-		2/1/-/-		**5**	**6**		
	Sprout	2^15^	*In vitro*	2/-/-													**2**			
		2^16^	LA	1/-/-									1/-/-		-/1/2/-		**2**	**1**	**2**	
	Leaves/Flower	3^17^	LA	3/-/-									2/-/-		-/3/3/-		**5**	**3**	**3**	
		1^18^	OA													D				
	Herb	1^19^	OA													E				
	Straw	2^20^	Ruminant												5/1/-/-		**5**	**1**		
**Total effects**				**13/2/-**	**-**	**1/1/-**	**2/2/-**	**-**	**-**	**2/3/-**	**-**	**-**	**18/7/-**		**24/57/11/-**		**60**	**72**	**12**	**-**
*Fagopyrum tataricum* (L.) Gaertn.	Grain/Seed	2^21^	*In vitro*							4/-/-							**4**			
		4^22^	LA	5/-/-					3/-/-	3/-/-			2/-/-		1/1/1/-		**14**	**1**	**1**	
	n.a	1^23^	*In vitro*	1/-/-					1/-/-								**2**			
		2^24^	OA							2/-/-			-/2/-				**2**	**2**		
		5^25^	LA	1/-/-					-/1/-	3/-/-			2/-/-		-/5/-/-		**6**	**6**		
	Sprout	1^26^	*In vitro*	1/-/-													**1**			
	Aerial parts	1^27^	LA	2/-/-					2/-/-								**4**			
	Bran	1^28^	Ruminant	1/-/-											3/-/-/-		**4**			
		2^29^	LA	1/-/-						-/1/-			1/1/-		2/1/-/-		**4**	**3**		
**Total effects**				**12/-/-**	**-**	**-**	**-**	**-**	**6/-/-**	**12/1/-**	**-**	**-**	**5/3/-**		**6/7/1/-**		**41**	**12**	**1**	**-**
*Polygonum aviculare* L.	Aerial part	4^30^	*In vitro*			3/-/-									4/-/-/-	F	**7**			
		3^31^	LA	1/-/-									-/1/-		-/1/1/-		**1**	**2**	**1**	
	Leaves/Flower	2^32^	*In vitro*			4/1/-		4/-/-									**8**	**1**		
		1^33^	LA													G				
	Herbs	2^34^	*In vitro*		-/1/-					1/-/-							**1**	**1**		
	n.a	4^35^	*In vitro*			2/-/-		2/-/-		1/-/-							**5**			
		1^36^	LA						1/-/-	1/-/-					1/-/-/-		**3**			
	Stem	1^37^	*In vitro*			4/-/-		4/-/-									**8**			
	Whole plant	1^38^	*In vitro*			1/-/-											**1**			
		1^39^	OA												-/2/-/-	H		**2**		
	Root	1^40^	OA							1/-/-							**1**			
**Total effects**				**1/-/-**	**-/1/-**	**14/-/-**	**-**	**10/-/-**	**1/-/-**	**4/-/-**	**-**	**-**	**-/1/-**		**5/3/1/-**		**35**	**6**	**1**	**-**
*Polygonum bistorta* L.	Aerial parts	3^41^	*In vitro*												4/-/-/-	I	**4**			
	Fruits	1^42^	*In vitro*		1/-/-					1/-/-							**2**			
	Herbs	1^43^	*In vitro*		-/1/-													**1**		
	N.a	1^44^	*In vitro*									1/-/-					**1**			
		1^45^	OA												1/1/-/-	H	**1**	**1**		
	Rhizome/Root/Tuber	4^46^	*In vitro*		1/-/-	2/-/-				2/2/-		1/-/-				J/K	**6**	**2**		
		2^47^	LA	1/-/-						1/-/-		1/-/-					**3**			
**Total effects**				**1/-/-**	**2/1/-**	**2/-/-**	**-**	**-**	**-**	**4/2/-**	**-**	**3/-/-**	**-**		**5/1/-/-**		**17**	**4**	**-**	**-**
*Polygonum hydropiper* L.	Aerial part	3^48^	*In vitro*			6/-/-		6/-/-							4/-/-/-		**16**			
	Herbs	2^49^	*In vitro*		-/1/-			4/-/-									**4**	**1**		
	Leaves	2^50^	*In vitro*			2/-/-				1/-/-							**3**			
	n.a	1^51^	*In vitro*					1/-/-									**1**			
	Stem/Stalks	1^52^	*In vitro*			2/-/-											**2**			
		1^53^	LA	1/-/-						1/-/-							**2**			
	Whole plant	1^54^	*In vitro*			1/-/-											**1**			
		1^55^	LA								3/-/-						**3**			
**Total effects**				**1/-/-**	**-/1/-**	**11/-/-**	**-**	**11/-/-**	**-**	**2/-/-**	**3/-/-**	**-**	**-**		**4/-/-/-**		**32**	**1**	**-**	**-**
*Polygonum minus* Huds.	Aerial part	1^56^	*In vitro*							1/-/-							**1**			
		1^57^	LA							1/-/-							**1**			
	Leaves	3^58^	*In vitro*			1/-/-		2/2/-								L	**3**	**2**		
		2^59^	LA	1/-/-						2/-/-						M	**3**			
**Total effects**				**1/-/-**	**-**	**1/-/-**	**-**	**2/2/-**	**-**	**4/-/-**	**-**	**-**	**-**		**-**		**8**	**2**	**-**	**-**
*Polygonum persicaria* L.	Aerial parts	2^60^	*In vitro*			1/-/-		4/1/-									**5**	**1**		
	Herbs	1^61^	*In vitro*		-/1/-													**1**		
**Total effects**				**-**	**-/1/-**	**1/-/-**	**-**	**4/1/-**	**-**	**-**	**-**	**-**	**-**		**-**		**5**	**2**	**-**	**-**
*Rheum officinale/ palmatum* L.	n.a	2^62^	*In vitro*			1/-/-				1/-/-						N	**2**			
		3^63^	LA						1/-/-	2/-/-						O	**3**			
	Rhizome/Root	10^64^	*In vitro*	1/-/-	1/-/-	3/-/-	-/-/1	1/-/-		-/2/-					2/-/-/-	P	**8**	**2**	**1**	
		1^65^	LA						-/1/-									**1**		
		2^66^	OA												-/2/-/-			**2**		
**Total effects**				**1/-/-**	**1/-/-**	**4/-/-**	**-/-/1**	**1/-/-**	**1/1/-**	**3/2/-**	**-**	**-**	**-**		**2/2/-/-**		**13**	**5**	**1**	**-**
*Rumex acetosa* L.	Aerial parts	3^67^	*In vitro*			1/-/-		2/-/-									**3**			
	Herbs	1^68^	*In vitro*			1/2/-											**1**	**2**		
	Root	1^69^	*In vitro*			3/-/-											**3**			
	Whole plant	2^70^	*In vitro*							2/-/-						Q	**2**			
		1^71^	OA																	
		2^72^	LA							2/-/-						R	**2**			
	Leaves	1^73^	LA												-/-/-/1					**1**
**Total effects**				**-**	**-**	**5/2/-**	**-**	**2/-/-**	**-**	**4/-/-**	**-**	**-**	**-**		**-/-/-/1**		**11**	**2**	**-**	**1**
*Rumex acetosella* L.	n.a	1^74^	*In vitro*			2/-/-		2/-/-									**4**			
	Whole plant	1^75^	*In vitro*			-/3/-												**3**		
	Root	1^76^	LA						1/-/-				/-/-		1/-/-/-		**3**			
**Total effects**				**-**	**-**	**2/3/-**	**-**	**2/-/-**	**1/-/-**	**-**	**-**	**-**	**1/-/-**		**1/-/-/-**		**7**	**3**	**-**	**-**
*Rumex crispus* L.	Aerial parts	2^77^	*In vitro*			4/1/-		-/3/-									**4**	**4**		
		1^78^	LA							-/2/-	-/2/-							**4**		
	Herbs	1^79^	*In vitro*			1/2/-											**1**	**2**		
	Leaves	3^80^	*In vitro*			6/4/-		4/3/-									**10**	**7**		
	n.a	1^81^	*In vitro*							1/-/-							**1**			
	Root	3^82^	*In vitro*			9/1/-		5/2/-									**14**	**3**		
	Seed	1^83^	*In vitro*			1/2/-		-/3/-									**1**	**5**		
**Total effects**				**-**	**-**	**21/10/-**	**-**	**9/11/-**	**-**	**1/2/-**	**-/2/-**	**-**	**-**		**-**		**31**	**25**	**-**	**-**
*Rumex obtusifolius* L.	Herbs	2^84^	*In vitro*			1/2/-									-/-/1/-		**1**	**2**	**1**	
	Leaves	1^85^	*In vitro*					1/-/-									**1**			
	n.a	2^86^	Ruminants																	
	Root	2^87^	*In vitro*			1/2/-				1/-/-							**2**	**2**		
		1^88^	LA							1/-/-							**1**			
	Seed	2^89^	*In vitro*			3/-/-										S	**3**			
	Whole plant	3^90^	Ruminants												1/-/-/-		**1**			
**Total effects**				**-**	**-**	**5/4/-**	**-**	**1/-/-**	**-**	**2/-/-**	**-**	**-**	**-**		**1/-/-/1**		**9**	**4**	**-**	**-**
*Rumex patientia* L.	Leaves/Flower	3^91^	*In vitro*			2/5/-		1/-/-									**3**	**5**		
	Root	2^92^	*In vitro*			4/-/-											**4**			
		2^93^	LA							8/1/-							**8**	**1**		
	Stem	1^94^	*In vitro*			1/-/-		1/-/-									**2**			
	Fruits	1^95^	LA							1/-/-							**1**			
**Total effects**				**-**	**-**	**7/5/-**	**-**	**2/-/-**	**-**	**9/1/-**	**-**	**-**	**-**		**-**		**18**	**6**	**-**	**-**
**Other plant species (n = 36)**		**25**^96^	**All groups**	**-**	**1/2/-**	**29/19/-**	**-**	**6/2/-**	**1/-/-**	**14/3/-**	**5/-/-**	**4/2/-**	**-**		**3/-/-/-**		**63**	**28**	**-**	**-**
**Total effects of all Polygonaceae species**				**30/2/-**	**4/6/-**	**103/45/-**	**2/2/1**	**50/16/-**	**10/2/-**	**61/14/-**	**8/2/-**	**7/2/-**	**24/11/-**	**51/70/14/1**			**356**	**172**	**15**	**1**

p (positive) = a positive effect, no (none) = no effect, n (negative) = a negative effect, question mark (?) = inconsistent effect; *In vivo*: Ruminant, labor animals (LA), other animals (OA); detailed information about the scoring procedure could be found in Table 3.

Other effects A: Cytotoxic effect, B: Modulatory effect of satiety hormones (DM2), C/H: Cytoprotective effect; modulatory effect on microorganisms, D: Stimulated growth of E. Ramulus, E: Supportive treatment of CVI, F: Antiobesity effect, G: Effective for kidney stones, I/K/L/Q: No toxic effect, J: Antispasmodic effect, M: Laxative effect, N: No wound healing, O: Smooth muscle reactivity, P: Spasmogenic effect, R: Antibiotic modulatory effect

Other plant species contain all plants with less than three publications: *Fallopia aubertii* (L.Henry) Holub, *Fallopia convolvulus* (L.) A. Löve (seed), *Fallopia dumetorum* (L.) Holub, *Oxyria digyna* (L.) Hill, *Polygonum alpinum* All., *Polygonum amphibium* L. (herb, whole plant), *Polygonum arenastrum* Boreau (leaves, stem), *Polygonum capitatum* D.Don (whole plant), *Polygonum lapathifolium* L. (herb, root/rhizome), *Polygonum mite* Schrank (herb), *Polygonum nepalense* Meisn., *Polygonum orientale* L. (aerial parts), *Polygonum perfoliatum* L. (herb, whole plant), *Polygonum polystachyum* Meisn., *Polygonum rurivagum* Boreau, *Polygonum viviparum* L., *Reynoutria x bohemica* Chrtek & Chrtkova (rhizome), *Reynoutria japonica* Houtt (rhizome)., *Reynoutria sachalinensis* (F. Schmidt) Nakai (rhizome), *Rheum rhabarbarum* L. (root), *Rumex x pratensis* Mert. & W.W.J. Koch, *Rumex alpestris* Jacq., *Rumex alpinus* L. (aerial part, flower, leaves, root), *Rumex aquaticus* L. (herb, root), *Rumex conglomeratus* Murray (herbs), *Rumex hydrolapathum* Huds. (leaves, root), *Rumex longifolius* DC., *Rumex maritimus* L. (root), *Rumex nebroides* Campd., *Rumex nivalis* Hegetschw., *Rumex palustris* Sm., *Rumex pulcher* (L.), *Rumex sanguineus* L. (whole plant), *Rumex scutatus* L. (whole plant), *Rumex* sp.*, Rumex thasiflorus* Fingerh. (herb, root)

1 (Amelchanka et al., 2010)

2 (Amelchanka et al., 2010; Kälber et al., 2013, 2011, 2012)

3 (Pulkrabek et al., 2017)

4 (Amelchanka et al., 2010; Hur et al., 2011; Metzger et al., 2007; Leiber et al., 2012; Paśko et al., 2019; Sinz et al., 2019; Swiatecka et al., 2013)

5 (Amelchanka et al., 2010)

6 (Choi et al., 2007; Lee et al., 2014; Zduńczyk et al., 2006; Kim et al., 2012, 2012; Fotschki et al., 2020; Préstamo et al., 2003; Tomotake et al., 2006)

7 (Leiber et al., 2009; Chowdhury & Koh, 2018a, b, 2019; Stringer et al., 2013; Sayed et al., 2015)

8 (Cui et al., 2020)

9 (Hosaka et al., 2014; Reeves et al., 2005; Mukoda et al., 2001)

10 (Benvenuti et al., 2012; Gāliņa et al., 2020; Gāliņa & Valdovska 2017; Flis et al., 2010)

11 (Coman et al., 2013; Sirotkin et al., 2021; Wolter et al., 2014; Yao et al., 2020)

12 (Keles et al., 2018; Wang et al., 2009a, 2008)

13 (Han et al., 2019; Huang et al., 2020; Kayashita et al., 1995, 1996, 1999; Li et al., 2020; Tomotake et al., 2001)

14 (Bijlani et al., 1985; Dong et al., 2019; Fang et al., 2007; Imaki et al., 1990; Jacob et al., 2008; Jacob & Carter 2008; Mišan et al., 2017; Wieslander et al., 2011, 2007)

15 (Lee et al., 2013; Liu et al., 2008)

16 (Watanabe & Ayugase 2010; Yang et al., 2016)

17 (Sadauskiene et al., 2018; Ðurendić-Brenesel et al., 2013; Lee et al., 2010)

18 (Simmering et al., 2002)

19 (Ihme et al., 1996)

20 (Li et al., 2020; Mu et al., 2019)

21 (Choi et al., 2017, 2015)

22 (Jin et al., 2020; Yan et al., 2012; Lee et al., 2013; Zhou et al., 2019b)

23 (Yang et al., 2020)

24 (Wieslander et al., 2011, 2007)

25 (Han et al., 2019; Kim et al., 2019; Zhou et al., 2018, 2020, 2019a)

26 (Liu et al., 2008)

27 (Yang et al., 2020)

28 (Zhao et al., 2017)

29 (Peng et al., 2020; Wang et al., 2009b)

30 (Sassi et al., 2007; Macheboeuf et al., 2014, Niderkorn & Macheboeuf, 2014; Sung et al., 2013)

31 (Milan et al., 2011; Sung et al., 2013)

32 (Proestos et al., 2008; Salama & Marraiki, 2010)

33 (Saremi et al., 2018)

34 (Smolarz & Skwarek 1999; Tunón et al., 1995)

35 (Akgunlu et al., 2016; Luo et al., 2018; Park et al., 2018; Seo et al., 2016)

36 (Park et al., 2018)

37 (Salama & Marraiki 2010)

38 (Gou et al., 2011)

39 (Youn & Noh, 2001)

40 (Begné et al., 2001)

41 (Macheboeuf et al., 2014; Niderkorn & Macheboeuf, 2014; Pirvu et al., 2017)

42 (Smolarz & Skwarek, 1999)

43 (Smolarz & Skwarek, 1999)

44 (Yu et al., 2015)

45 (Khan et al., 2008)

46 (Ali et al., 2015; Pawłowska et al., 2020; Smolarz & Skwarek, 1999; Liu et al., 2014)

47 (Ali et al., 2015; Khushtar et al., 2018)

48 (Ayaz et al., 2016; Macheboeuf et al., 2014; Niderkorn & Macheboeuf, 2014)

49 (Liu et al., 2012; Smolarz & Skwarek, 1999)

50 (Nasir et al., 2020; Yang et al., 2012)

51 (Zhang et al., 2020)

52 (Nasir et al., 2020)

53 (Zhang et al., 2018)

54 (Lee & Vairappan, 2011)

55 (Rahman et al., 2002)

56 (George et al., 2014)

57 (George et al., 2014)

58 (Jamal et al., 2011; Mackeen et al., 1997; Qader et al., 2011)

59 (Qader et al., 2012; Wasman et al., 2010)

60 (Derita & Zacchino, 2011; Hussain et al., 2010)

61 (Smolarz & Skwarek, 1999)

62 (Tsai et al., 2004; Yang et al., 2017)

63 (Yang et al., 2017; Ji et al., 2020; Zhang et al., 2015a)

64 (Bae et al., 1998; Zhang et al., 2011; García-González et al., 2008a, b; Yu et al., 2005; Goto et al., 1996; Blaszczyk et al., 2000; Kosikowska et al., 2010; Nanasombat et al., 2018; Lin et al., 2009)

65 (Wang et al., 2011)

66 (Wei et al., 2021; Straub et al., 2005)

67 (Johann et al., 2008; Johann et al., 2010; Schmuch et al., 2015)

68 (Orbán-Gyapai et al., 2017)

69 (Orbán-Gyapai et al., 2017)

70 (Bae et al., 2012; Hussain et al., 2015)

71 (Hussain et al., 2015)

72 (Bae et al., 2012; Hussain et al., 2015)

73 (Ladeji et al., 1997)

74 (Akgunlu et al., 2016)

75 (Orbán-Gyapai et al., 2017)

76 (Alkushi, 2017)

77 (Coruh et al., 2008; Ulukanli et al., 2005)

78 (Kozan et al., 2006)

79 (Orbán-Gyapaiet al., 2017)

80 (Yıldırım et al., 2001; Idris et al., 2019; Orbán-Gyapai et al., 2017)

81 (Pan et al., 2020)

82 (Idris et al., 2019; Orbán-Gyapai et al., 2017; Ulukanli et al., 2005)

83 (Yıldırım et al., 2001)

84 (Orbán-Gyapai et al., 2017; Purcell et al., 2012)

85 (Bineshian et al., 2019)

86 (Derrick et al., 1993; Wilman et al., 1997)

87 (Orbán-Gyapai et al., 2017; Singh & Purohit, 2018)

88 (Singh & Purohit, 2018)

89 (Ginovyan et al., 2020; Ginovyan & Trchounian, 2019)

90 (Waghorn & Jones, 1989; Derrick et al., 1993; Molan et al., 2007)

91 (Orbán-Gyapai et al., 2017; Ceylan et al., 2019)

92 (Jeppesen et al., 2012; Orbán-Gyapai et al., 2017)

93 (Süleyman et al., 1999, 2004)

94 (Ceylan et al., 2019)

95 (Gürbüz et al., 2005)

96 (Harrold et al., 1980; Smolarz & Skwarek, 1999; Özbay & Alim, 2009; Ceylan et al., 2019; Liao et al., 2011; Keleş et al., 2001; Smolarz & Skwarek, 1999; Gou et al., 2017; Saleem et al., 2018; Liu et al., 2012; Nawrot-Hadzik et al., 2019; Rehman et al., 2019; Ozturk & Ozturk, 2007; Orbán-Gyapai et al., 2017; Rouf et al., 2003)

As to single species, effects of *Fagopyrum esculentum* Moench were investigated the most (144 times, with 131 *in vivo* and 13 *in vitro* experiments). The second most frequently studied species was *Rumex crispus* L. (56 times, with 52 *in vivo* and 4 *in vitro* experiments) followed by *Fagopyrum tataricum* (L.) Gaertn. (54 times, including 47 *in vivo* and 7 *in vitro* experiments), *Polygonum aviculare* L. (42 times; 10 *in vivo* and 32 *in vitro* experiments) and *Polygonum hydropiper* L. (33 times, with 5 *in vivo* and 28 *in vitro* experiments*).*

Most frequently, antibacterial effects were investigated for *Rumex crispus* L. (31 times) and *Polygonum aviculare* L. (15 times), anti-inflammatory effects for *Fagopyrum tataricum* (L.) Gaertn. (13 times) and *Rumex patientia* L. (10 times), antifungal effects for *Rumex crispus* L. (20 times) and *Polygonum hydropiper* L. (11 times), antihyperlipidemic effects for *Fagopyrum esculentum* Moench (25 times) and *Fagopyrum tataricum* (L.) Gaertn. (8 times), antioxidative effects for *Fagopyrum esculentum* Moench (15 times) and *Fagopyrum tatatricum* (L.) Gaertn. (12 times).

#### 3.2.2 Effects potentially relevant for livestock nutrition

Most frequently, effects on growth performance were studied (49 times), followed by effects on feed intake (39 times) and feed conversion rate (17 times; Table 5, Table 6). Effects on methane reduction (12 times), ammonia reduction (9 times), meat quality (6 times) and milk quality (4 times) were studied less frequently. With regard to specific species again most studies were dealing with *Fagopyrum esculentum* Moench (93 times containing 87 *in vivo* and 6 *in vitro* effects). A lower number of studies was dealing with *Fagopyrum tataricum* (L.) Gaertn. (14 times containing solely *in vivo*), *Polygonum aviculare* L. (9 times containing 5 *in vivo* and 4 *in vitro*) and *Polygonum bistorta* L. (6 times containing 2 *in vivo* and 4 *in vitro****)*.** These four Polygonaceae species were the most intensively researched species with regard to feeding effects in livestock.

**Table 5:** Potentially livestock-relevant effects of 11 Polygonaceae species.

Plant species	Plant parts	Number of publications	Type of experiments	Ammonia reduction	Methane reduction	Milk quality	Meat quality	Improved performance	Improved feed intake	Improved conversion rate	Total effects of individual species x plant part			
				p/no/n	p/no/n	p/no/n	p/no/n	p/no/n/?	p/no/n	p/no/n	p	no	n	?
*Fagopyrum esculentum* Moench	Whole plant	2^1^	*In vitro* (rumen content of cow)	1/1/-	1/1/-						**2**	**2**		
		4^2^	Cows	1/-/-		2/2/-		-/2/-/-	1/3/-		**4**	**7**		
		1^3^	Mice					-/1/-/-	-/1/-			**2**		
	Grain/Seed	3^4^	*In vitro* (rumen content of cow)	2/1/-	1/1/1						**3**	**2**	**1**	
		1^5^	Cows				-/1/-		-/1/-			**2**		
		7^6^	Mice, rats					1/6/2/-	1/6/-	2/1/-	**4**	**13**	**2**	
		5^7^	Broilers					1/2/-/-	2/1/-	1/1/1	**4**	**4**	**1**	
			Laying hen					-/1/-/-	1/-/-	-/1/-	**1**	**2**		
	Hull/Bran	2^8^	Mice, rats					-/2/-/-	-/1/-			**3**		
		4^9^	Pigs				-/1/-					**1**		
			Laying hen					1/-/-/-	1/-/-	-/1/-	**2**	**1**		
			Piglets				-/1/-	1/-/-/-	-/1/-		**1**	**3**		
	n.a	3^10^	Goat											
			Lambs				1/-/-	-/1/-/-	1/-/-	-/1/-	**2**	**2**		
		5^11^	Mice, rats					-/4/2/-	-/4/1	-/1/-		**9**	**3**	
		4^12^	Barrow											
			Broiler					-/1/-/-	1/-/-	1/-/-	**2**	**1**		
			Chicken											
			Turkey											
	Sprout	2^13^	Mice, rats					-/-/2/-	-/1/-			**1**	**2**	
	Leaves/Flower	2^14^	Rats					-/2/2/-	-/1/1	-/2/-		**3**	**3**	
	Straw	2^15^	Lambs				1/-/-	1/1/-/-	2/-/-	1/-/-	**5**	**1**		
**Total effects**				**2/1/-**	**-**	**2/2/-**	**2/3/-**	**5/20/8/-**	**9/20/2**	**4/9/1**	**24**	**57**	**12**	**-**
*Fagopyrum tataricum* (L.) Gaertn.	Grain/Seed	2^16^	Mice, Rats					1/-/1/-	-/1/-		**1**	**1**	**1**	
	n.a	3^17^	Mice, rats					-/3/-/-	-/2/-			**5**		
	Bran	1^18^	Lambs				1/-/-	1/-/-/-	1/-/-		**3**			
		2^19^	Rats					1/1/-/-	1/-/-		**2**	**1**		
**Total effects**				**-**	**-**	**-**	**1/-/-**	**3/4/1/-**	**2/3/-**	**-**	**6**	**7**	**1**	**-**
*Polygonum aviculare* L.	Aerial part	2^20^	*In vitro* (rumen content of sheep)	2/-/-	2/-/-						**4**			
		1^21^	Mice					-/-/1/-	-/1/-			**1**	**1**	
	n.a	1^22^	Mice					1/-/-/-			**1**			
	Whole plant	1^23^	Broiler					-/1/-/-		-/1/-		**2**		
**Total effects**				**2/-/-**	**2/-/-**	**-**	**-**	**1/1/1/-**	**-/1/-**	**-/1/-**	**5**	**3**	**1**	**-**
*Polygonum bistorta* L.	Aerial part	2^24^	*In vitro* (rumen content of sheep)	2/-/-	2/-/-						**4**			
	n.a	1^25^	Broiler					1/-/-/-		-/1/-	**1**	**1**		
**Total effects**				**2/-/-**	**2/-/-**	**-**	**-**	**1/-/-/-**	**-**	**-/1/-**	**5**	**1**	**-**	**-**
*Polygonum hydropiper* L.	Aerial part	2^26^	*In vitro* (rumen content of sheep)	2/-/-	2/-/-						**4**			
**Total effects**				**2/-/-**	**2/-/-**	**-**	**-**	**-**	**-**	**-**	**4**	**-**	**-**	**-**
*Rheum officinale/ palmatum* L.	Rhizome/root	2^27^	*In vitro* (rumen content of sheep)		2/-/-						**2**			
		1^28^	Piglets						-/1/-			**2**		
**Total effects**				**-**	**2/-/-**	**-**	**-**	**-/-/-/-**	**-/1/-**	**-**	**2**	**2**	**-**	**-**
*Rumex acetosa* L.	Whole plant	1^29^	Chicks											
	Leaves	1^30^	Rats					-/-/-/1						**1**
**Total effects**				**-**	**-**	**-**	**-**	**-/-/-/1**	**-**	**-**	**-**	**-**	**-**	**1**
*Rumex acetosella* L.	Root	1^31^	Rats					1/-/-/-			**1**			
**Total effects**				**-**	**-**	**-**	**-**	**1/-/-/-**	**-**	**-**	**1**	**-**	**-**	**-**
*Rumex obtusifolius* L.	herbs	1^32^	*In vitro* (rumen content of steer)		1/-/-						**1**			
	n.a	2^33^	Lambs											
			Sheep											
	Whole plant	1^34^	Lambs											
			Sheep											
**Total effects**				**-**	**1/-/-**	**-**	**-**	**-**	**-**	**-**	**1**	**-**	**-**	**-**
*Fallopia convolvulus* (L.) A. Löve	Seed	1^35^	Rats					1/-/-/-	1/-/-	1/-/-	**3**			
**Total effects**				**-**	**-**	**-**	**-**	**1/-/-/-**	**1/-/-**	**1/-/-**	**3**	**-**	**-**	**-**
**∑ total effects**				**8/1/-**	**9/2/1**	**2/2/-**	**3/-/-**	**12/26/10/1**	**12/25/2**	**5/11/1**	**58**	**72**	**14**	**1**

P (positive) = is a positive effect, no (none) = is no effect, n (negative) = a negative effect, question mark (?) = inconsistent effect; detailed information about the scoring procedure could be found in Table 3.

1 (Amelchankaet al., 2010; Leiber et al., 2012)

2 (Amelchanka et al., 2010; Kälber et al., 2013, 2012, 2011)

3 (Pulkrabek et al., 2017)

4 (Amelchanka et al., 2010; Sinz et al., 2019; Leiber et al., 2012)

5 (Amelchanka et al., 2010)

6 (Choi et al., 2007; Zduńczyk et al., 2006; Kim et al., 2012, 2012; Fotschki et al., 2020; Préstamo et al., 2003; Tomotake et al., 2006)

7 (Leiber et al., 2009; Chowdhury & Koh, 2018a, b, 2019; Sayed et al., 2015)

8 (Reeves et al., 2005; Mukoda et al., 2001)

9 (Benvenuti et al., 2012; Gāliņa et al., 2020; Gāliņa & Valdovska 2017; Flis et al., 2010)

10 (Keles et al., 2018; Wang et al., 2008, 2009a)

11 (Huang et al., 2020; Kayashita et al., 1996, 1999, 1995; Tomotake et al., 2001)

12 (Dong et al., 2019; Fang et al., 2007; Jacob & Carter, 2008; Jacob et al., 2008)

13 (Watanabe & Ayugase, 2010; Yang et al., 2016)

14 (Đurendić-Brenesel et al., 2013; Lee et al., 2010)

15 (Li et al., 2020; Mu et al., 2019)

16 (Jin et al., 2020; Zhou et al., 2019b)

17 (Kim et al., 2019; Zhou et al., 2018, 2019a)

18 (Zhao et al., 2017)

19 (Peng et al., 2020; Wang et al., 2009b)

20 (Macheboeuf et al., 2014; Niderkorn & Macheboeuf, 2014)

21 (Sung et al., 2013)

22 (Park et al., 2018)

23 (Youn & Noh, 2001)

24 (Macheboeuf et al., 2014; Niderkorn & Macheboeuf, 2014)

25 (Khan et al., 2008)

26 (Macheboeuf et al., 2014; Niderkorn & Macheboeuf, 2014)

27 (García-González et al., 2008a, b)

28 (Straub et al., 2005)

29 (Hussain et al., 2015)

30 (Ladeji et al., 1997)

31 (Alkushi, 2017)

32 (Purcell et al., 2012)

33 (Derrick et al., 1993; Wilman et al., 1997)

34 (Derrick et al., 1993)

35 (Harrold et al., 1980)

**Table 6:** Further information about potentially livestock-relevant effects of 8 Polygonaceae species.

Plant species	Plant parts	Number of publications	Type of experiments	Further information
*Fagopyrum esculentum* Moench	Whole plant	2^1^	*In vitro* (rumen content of cow)	Buckwheat forage and silage had no significant effect on *in vitro* ruminal degradability and short chain fatty acid concentration. Buckwheat forage enhanced estimated microbial Nitrogen growth efficiency. Buckwheat forage reduced the number of bacteria in the incubated fluid, while Buckwheat silage reduced that of holotrich protozoa significantly compared to control.
		4^2^	Cows	Transfer rate of a-linoleic acid from feed to milk was significantly higher than ryegrass, but there was no effect on alpha-tocopherol in milk. Ruminal Nitrogen balance was significantly lower, fecal Nitrogen loss and Nitrogen utilization for milk were significantly higher than ryegrass.
	Grain/Seed	3^4^	*In vitro* (rumen content of cow)	There was no effect on *in vitro* ruminal degradability and short chain fatty acid concentration. pH was significantly higher than wheat. Nitrogen supply, apparent degraded Nitrogen, degraded but not recovered Nitrogen, non-ammonia Nitrogen decreased significantly compared to wheat. Holotrich protozoa counts were significantly higher and total gas was significantly lower than wheat; There was no significant effect on total gas production and microbial count. Short chain fatty acid, Propionate, acetate concentration increased significantly compared to acacia.
		1^5^	Cows	There was no significant effect on milk quality and feed intake compared to the control diet.
		5^7^	Broilers	Ileal digestibility of Phytate phosphorus was significantly higher than control diet (0.15 % lower non-Phytate). Phytase activity was significantly higher, but decreased in digestive tract parts. Phytase activity was significantly lower in ileum. Bone quality (Tibia, Femur) and Phosphorus and Nitrogen retention were significantly higher, Total Phosphorus excretion was significantly lower than control diet (0.16 % lower non-Phytate).
			Laying hen	Egg production rate, egg quality and Phosphorus and Nitrogen retention were significantly higher than control diet (0.16 % lower non-Phytate). There was no significant effect on egg quality.
	Hull/Bran	4^9^	Pigs	There was no significant effect on meat oxidative stability compared to the control diet (some contain additionally oat or vitamin E).
			Laying hen	Egg production rate was significantly higher than control diet. There was no effect on egg quality. Buckwheat bran was preferred to soybean in the free-choice feeding test.
			Piglets	Mean corpuscular volume (MCV), mean corpuscular hemoglobin (MCH), alkaline phosphatase (ALP), Calcium (CA) levels were significantly lower in Probiotics + Buckwheat than only probiotics. There was no significant probiotic effect. Intestinal morphology, total number and density of goblet cells had no significant changes. Ki-67+ cells were significantly higher in colon. CD3+ cells were significantly lower in Jejunum and higher in Colon.
	n.a	3^10^	Goat	Phosphorus and Crude protein degradability of Buckwheat was comparable with other cereals. Starch degradability was slower than the other cereals.
			Lambs	There was no significant effect on Carcass parameters. Yellowness of M. longissimus dorsi of lambs was significantly lower, but the Total phenolic content was significantly higher than maize.
		4^12^	barrow	Apparent total tract digestibility of Organic matter was significantly higher and Neutral detergent fiber/Acid detergent fiber were significantly lower than control diet (Soybean). There was no significant effect on Energy balance and energy content. There was no significant effect on true and apparent Phosphorus digestibility.
			chicken	True metabolizable energy was lower compared to control (fasted): The content was higher compared to NRC.
			turkey	True metabolizable energy was lower compared to control (fasted): The content was higher compared to NRC.
	Straw	2^15^	Lambs	Dry matter digestibility and Nitrogen retention were higher, while Nitrogen excretion was lower than control diet. Nutrient digestibility was significantly higher than control diet. The ruminal microbial diversity was declined and the microbiome tends to be simplified and some maleficent bacteria abundance was increased.
*Fagopyrum tataricum* (L.) Gaertn.	Bran	1^18^	Lambs	There was no significant effect on serum parameters. Kidney, rumen and omasum weights were significantly lower, Reticulum and small intestine weights were significantly higher than basal diet.
*Polygonum aviculare* L.	Aerial part	2^20^	*In vitro* (rumen content of sheep)	30 % less methane production and 80 % less ammonia production than ryegrass. True organic matter digestibility, crude protein, gas production, volatile fatty acid were significantly lower than ryegrass.
	Whole plant	1^23^	Broiler	There was no significant effect on bloody diarrhea, survival, lesion score. Oocyst excretion was lower than model group (infected). There was no significant anticoccidial effect.
*Polygonum bistorta* L.	Aerial part	2^24^	*In vitro* (rumen content of sheep)	30 % less methane production and 80 % less ammonia production than ryegrass. True organic matter digestibility, gas production, volatile fatty acid were significantly lower than ryegrass. Crude protein was significantly higher than ryegrass.
	n.a	1^25^	Broiler	Serum Glucose and total protein were significantly higher, Urea nitrogen was significantly lower than model group (infected). Carcass fat weight and grid reference tissue depth increased while drip loss decreased. There was an anticoccidial activity.
*Polygonum hydropiper* L.	Aerial part	2^26^	*In vitro* (rumen content of sheep)	30 % less methane production and 80 % less ammonia production than ryegrass. True organic matter digestibility, crude protein, gas production, volatile fatty acid were significantly lower than ryegrass.
*Rheum officinale/ palmatum* L.	Rhizome/root	2^27^	*In vitro* (rumen content of sheep)	Dry matter digestibility, neutral detergent fiber digestibility, total gas production, total volatile fatty acid production decreased significantly compared to control; Dry matter digestibility, cell wall digestibility, total gas production, total volatile fatty acid production decreased significantly compared to control.
		1^28^	Piglets	Fecal N loss and energy digestibility and metabolizability were significantly higher, fecal energy loss was significantly lower than basal diet. There was no significant effect on nitrogen and energy balance. Dry matter content of the feces was decreased linearly to the increasing amount of rhubarb. Higher dose of rhubarb (1 %) caused a laxative effect.
*Rumex acetosa* L.	Whole plant	1^29^	Chicks	Coppersulphate, brassica compestris and cisplatin induced emesis were significantly decreased compared to negative control.
*Rumex obtusifolius* L.	n.a	2^33^	Lambs	Mineral content of rumen and feces and mineral availability changed. The dock particles in the rumen typically had a low ratio of length to width and it seemed that dock particles did not need to be reduced in size as much as ryegrass particles before passing out of the rumen. Fibrosity index, time spent ruminating and time spent eating was lower than ryegrass.
			Sheep	Mineral content in rumen changed. Fibrosity index was lower than ryegrass.
	Whole plant	1^34^	Lambs	Feed intake and Body weight gain were lower compared to ryegrass. Organic matter digestibility was lower and dry matter digestibility was higher than ryegrass. Dry weight in rumen was lower and fecal loss of rumen content was higher than ryegrass.
			Sheep	Dry matter digestibility was higher and organic matter digestibility was lower than ryegrass.

1 (Amelchankaet al., 2010; Leiber et al., 2012),

2 (Amelchanka et al., 2010; Kälber et al., 2013, 2012, 2011),

4 (Amelchanka et al., 2010; Sinz et al., 2019; Leiber et al., 2012),

5 (Amelchanka et al., 2010),

7 (Leiber et al., 2009; Chowdhury & Koh, 2018a, b, 2019; Sayed et al., 2015)

9 (Benvenuti et al., 2012; Gāliņa et al., 2020; Gāliņa & Valdovska, 2017; Flis et al., 2010),

10 (Keles et al., 2018; Wang et al., 2008a, 2009b),

12 (Dong et al., 2019; Fang et al., 2007; Jacob & Carter, 2008; Jacob et al., 2008),

15 (Li et al., 2020; Mu et al., 2019),

18 (Zhao et al., 2017),

20 (Macheboeuf et al., 2014; Niderkorn & Macheboeuf, 2014),

23 (Youn & Noh, 2001),

24 (Macheboeuf et al., 2014; Niderkorn & Macheboeuf, 2014),

25 (Khan et al., 2008),

26 (Macheboeuf et al., 2014; Niderkorn & Macheboeuf, 2014),

27 (García-González et al., 2008a, b),

28 (Straub et al., 2005),

29 (Hussain et al., 2015),

33 (Derrick et al., 1993; Wilman et al., 1997),

34 (Derrick et al., 1993).

Specifically, effects on growth performance were investigated most frequently (*Fagopyrum esculentum* Moench (33 times), and *Fagopyrum tataricum* (L.) Gaertn. (8 times)), followed by effects on feed intake (*Fagopyrum esculentum* Moench (31 times), and *Fagopyrum tataricum* (L.) Gaertn. (5 times)), and effects on feed conversion rate (*Fagopyrum esculentum* Moench (14 times)), effects on milk and meat quality (*Fagopyrum esculentum* Moench (9 times)), and effects on ammonia and methane reduction (*Fagopyrum esculentum* Moench (6 times)).

### 3.3 Outcome of published evidence regarding veterinary, pharmacological and/or biological effects

For 33 of the investigated 49 Polygonaceae species, publications reporting on pharmacological and/or biological effects were found. No studies on immunomodulatory, antitussive and astringent effects were identified, and only sparse information on antiviral, antiparasitic, prebiotic, probiotic, modulatory, antidiarrheal, hepatoprotective and antinociceptive/ analgesic effects was found. Studies were predominantly dealing with antibacterial, anti-inflammatory, antifungal, antioxidative and antihyperlipidemic properties (Table 4). The investigated Polygonaceae species showed some *in vitro* antibacterial activity against numerous gram positive (*Streptococcus pyogenes, Enterococcus faecalis, Staphylococcus aureus, Staphylococcus epidermidis, Staphylococcus saprophyticus, Bacillus subtilis*) and gram negative bacteria (*Escherichia coli, Klebsiella pneumoniae, Pseudomonas aerigunosa, Neisseria gonorrhoeae, Salmonella typhi, Salmonella paratyphi, Salmonella enteriditis, Shigella flexneri, Achinetobacter baumanii*) (Additional file 3). Since *E.coli*-induced digestive disorders are frequently observed in the postweaning period of piglets, Polygonaceae species might be useful for this treatment (Madec et al., 1998).

Numerous *in vitro* experiments indicated antifungal properties of the Polygonaceae species, with activity against a wide spectrum of fungi (*Aspergillus flavus, Aspergillus fumigatus, Aspergillus niger, Cryptococcus neoformans, Candida albicans, Candida krusei, Microsporum gypseum, Tychophyton rubrum, Trychophyton mentagophytes, Saccharomyces cerevisiae, Alternia solani, Harpophora maydis*; Additional file 3). Given that livestock diseases like cattle ringworm may be caused by *T. mentagophytes* (Rook & Frain-Bell, 1954), some Polygonaceae species may be useful as a supportive treatment.

Many *in vitro* and *in vivo* experiments were dealing with anti-inflammatory activity of Polygonaceae species. On a mechanistic level a decrease of proinflammatory cytokines, such as tumor necrosis factor-a (TNF-a), interleukins (IL) and lipopolysaccharides (LPS), or the inhibition of cyclooxygenases were reported. Activity against ethanol-induced gastric ulcers and inhibition of carrageenan- and xylene-induced edema were described. The anti-inflammatory activity may be particularly useful for diseases in young animals, such as respiratory or gastrointestinal diseases (Ayrle et al., 2016). Polygonaceae species are rich in phenolic compounds and therefore act as antioxidants. Beside the well known direct radical scavenging activity of polyphenols, the experimental shown antioxidative activity is also based on increasing levels of antioxidant enzyme activities, such as superoxide dismutase (SOD), glutathione peroxidase (GPX) and catalase (CAT), which are important for detoxifying of free radicals.

Fagopyrum species are interesting with regard to their anti-inflammatory, antioxidative and antihyperlipidemic activities. The genera “Polygonum” and “Rumex” show mainly antibacterial, antifungal and anti-inflammatory activities. In addition, antioxidative properties are shown by Polygonum species and antihyperlipidemic properties in Rumex species. Few experiments were conducted with the genus “Rheum”, which showed antioxidative, antibacterial, antifungal and anti-inflammatory activities.

Thus, *Polygonum* and *Rumex* species may be useful in the case of bacterial and fungal infections. Species from almost all genera exhibited some anti-inflammatory activity and may thus be useful to mitigate inflammatory ailments. On the other hand, only *Fagopyrum* species showed a pronounced cholesterol-lowering activity.

Some Polygonaceae species (17 species, *e.g. Polygonum aviculare* L., *Rumex crispus* L. and *Rumex obtusifolius* L.) seem to play an important role in European ethnoveterinary medicine and are commonly used for the treatment of disorders of the gastrointestinal tract and metabolism, and skin diseases (Table 1). However, for 32 species no ethnoveterinary use has been documented yet.

Data only from *in vitro* experiments or *in vivo* experiments with laboratory animals have been reported for *Polygonum minus* Huds., *Polygonum persicaria* L., *Rumex crispus* L. and *Rumex patienti*a L., but without a link to livestock relevant diseases (Table 4). Extracts of *Polygonum minus* Huds. showed anti-inflammatory properties. The plant contains phenolic compounds (Qader et al., 2012). No ethnoveterinary uses have been reported for *Polygonum minus* Huds. Extracts of *Polygonum persicaria* L. showed antifungal properties *in vitro* against a wide range of fungi (Additional file 3). The plant contains compounds like sesquiterpenes and flavonoids (Derita & Zacchino, 2011). The ethnoveterinary use of *Polygonum persicaria* L. in case of genitourinary diseases has so far not been corroborated by *in vitro* and/or *in vivo* studies (Mayer et al., 2014). Extracts of *Rumex crispus* L. showed activity against a wide range of gram positive and negative bacteria, and fungi (Additional file 3), supporting the ethnoveterinary use for the treatment of skin and gastrointestinal diseases (Mayer et al., 2014). Extracts of *Rumex patientia* L. showed antibacterial and anti-inflammatory activities (Additional file 3). Also, such extracts were effective *in vivo* against gastric ulcers (Süleyman et al., 2004) and inhibited carrageenan-induced edema (Süleyman et al., 1999). There are no reported ethnoveterinary uses for *Rumex patientia* L. Both. *Rumex crispus* L. and *Rumex patientia* L. contain phenolic compounds, such as anthraquinones, flavonoids and stilbenes (Orbán-Gyapai et al., 2017).

### 3.4 Specific livestock nutrition-related properties of Polygonaceae species

Plants relevant for various livestock animals are predominantly the cultivated Polygonaceae species, in particular buckwheat which is used as poultry feed (Jacob & Carter, 2008; Leiber, 2016) but also considered as feed for pigs (Dong et al., 2019; Farrell, 1978) and a concentrate or forage for ruminants (Leiber, 2016; Mu et al., 2019; Omokanye et al., 2021). Rhubarb (mainly roots or rhizomes) is considered as an alternatives to antibiotic growth promoters in pig diets (Straub et al., 2005). Of the wild Polygonaceae abundant in Europe, mainly *Polygonum* species occurring in natural pastures are mentioned in the literature as feed selected by grazing ruminants (Leps et al., 1995; Krahulec et al., 2001; Gorlier et al., 2012; Kazmin et al., 2016). However, also *Rumex* species, which are almost exclusively considered as weeds, are eaten by ruminants (Hejcman et al., 2014; Sormunen-Cristian et al., 2012; Waghorn & Jones, 1989; Zaller, 2006). For buckwheat, interesting effects on rumen fermentation are reported, which affect fatty acid composition of the products (Kälber et al., 2011), as well as methane formation (Leiber et al., 2012) and ammonia levels (Kälber et al., 2012). Rumen-modulating effects were also shown for rhubarb roots (García-González et al., 2008b; Kim et al., 2016). *Rumex obtusifolius* may have effects against bloating (Waghorn & Jones, 1989) and affects ruminal protein degradation and growth of proteolytic bacteria (Molan et al., 2007).

For 11 of the investigated 49 Polygonaceae species, 66 publications on effects potentially relevant for animals nutrition and performance were found (Table 5, Table 6). Growth performance, milk yield, egg yield, feed intake and feed conversion rate were investigated in cows, mice, rats, chicken, pigs, goats, lambs, barrows, broiler and turkey. The majority of these publications showed no effects on feed intake, growth performance and feed conversion rate. Of the few effects reported, positive effects predominated over negative properties. Thus, some Polygonaceae species may improve the above parameters to some extent. The effects on the quality of milk, meat and eggs were either positive or absent.

The plant most frequently studied in controlled feeding experiments was buckwheat. A total of 12 ruminant experiments with *Fagopyrum esculentum*, and one with *Fagopyrum tataricum* have been reported. Buckwheat was the only species for which negative effects on feed intake and performance were never reported (except for rodents). Some positive effects were found, such as an improved ruminal or overall N conversion, lower methanogenesis, and positive effects on milk or meat quality. Three studies (four experiments) considering roots of *Rheum* spp. as a feedstuff for farm animals were reported, showing *in vitro* mitigation of ruminal methanogenesis but no effects on performance of piglets *in vivo*. We found four experiments with *Rumex obtusifolius* in which lowering of methane production and inhibition of proteolytic bacteria was shown *in vitro*, and prevention of bloating *in vivo. Polygonum bistorta* is the most abundant species of Polygonaceae in pastures, and is clearly selected by ruminants (Leps et al., 1995; Krahulec et al., 2001; Gorlier et al., 2012). Two *in vitro* experiments with this species revealed mitigation of ruminal methane and ammonia formation.

Feeding buckwheat, rhubarb or Polygonaceae has modulating effects on the rumen processes. This is evident from *in vivo* and *in vitro* experiments where either methane and ammonia production or fatty acid profiles were taken into account.

### 3.5 Polygonaceae species most relevant for veterinary treatment and nutrition of farm animals

#### 3.5.1 *Fagopyrum esculentum* Moench *and Fagopyrum tataricum (L.)* Gaertn.

In experiments with both *Fagopyrum* species, sprouts, leaves, stems and flowers, but mainly the seeds, hull and bran and whole plants were investigated. For both species *in vivo* and *in vitro* experiments showed their antioxidative activity. In addition, *Fagopyrum esculentum* Moench (grains and seeds, hulls and brans, sprouts, leaves and flowers) showed antihyperlipidemic, and the grains and seed of *Fagopyrum tataricum* (L.) Gaertn anti-inflammatory activity (Table 4). Flavonoids and other phenolics are known to possess antioxidant properties (Đurendić-Brenesel et al., 2013; Flis et al., 2010; Jin et al., 2020), while flavonoids of *Fagopyrum esculentum* Moench may be responsible for the antihyperlipidemic activity. Buckwheat seeds could contribute to the improvement of the lipid profile in serum and liver (Choi et al., 2007). Seeds of *Fagopyrum tataricum* (L.) Gaertn. are rich in rutin which may explain, in part, their anti-inflammatory activity (Choi et al., 2015). Gastrointestinal diseases with inflammatory manifestation are a major challenge in piglets (Ayrle et al., 2016), and *Fagopyrum tataricum* (L.) Gaertn. may be useful in the treatment of postweaning diarrhea. Antioxidant activity of buckwheat hulls and brans were reported in pigs (Flis et al., 2010). Administration of buckwheat hulls and brans lowered the Enterobacteriaceae count in piglets and improved growth performance in the adaptive phase of postweaning (Gāliņa & Valdovska, 2017). One of the most important diseases of dairy cows are disturbance of fat and liver metabolism in early lactation (Durrer et al., 2020). The antihyperlipidemic activities of *Fagopyrum esculentum* Moench might be of interest in this context. However, phototoxic fagopyrins in Fagopyrum species may limit feedable amounts (Benković et al., 2014). Although *Fagopyrum esculentum* Moench is reported in ethnoveterinary sources for its fertility (Vogl et al., 2016) purposes, no *in vivo* or *in vitro* data on this use could be found in the recent literature.

The above-mentioned phenolic compounds (mainly Rutin; Leiber et al., 2012) are essentially involved in the modulating effects of buckwheat seeds and whole plant on rumen processes. It was shown that dietary buckwheat can lower methane formation. Also, ammonia production was lowered, as shown by *in vivo* and *in vitro* studies (Table 5; Sinz et al., 2019). Thus could imply a lesser burden to the liver of ruminants (Parker et al., 1995), lower metabolic urea levels, and a better utilization of dietary nitrogen (Kälber et al., 2012; Kapp-Bitter et al., 2023) resulting in lower urinary nitrogen emissions. A higher transfer rate of omega-3 fatty acids from feed to milk in cows supplemented with buckwheat (Kälber et al., 2011), are possibly due to a protective effect in the rumen of buckwheat polyphenols on these fatty acids (Buccioni et al., 2012). This is beneficial for the animal itself (Leiber et al., 2019) as well as for the consumer (Sinclair et al., 2002).

Numerous studies investigated the basic dietary properties of *Fagopyrum esculentum* Moench in cows, mice, rats, broilers, laying hens, pigs, piglets, goat, lambs, barrows, chicken and turkeys. In the majority of experiments no effects were observed on growth performance, feed intake and feed conversion rate. Nevertheless, some experiments show significant effects, with either improvement or impairment of intake and performance (Table 5). For all investigated farm animals, the feed properties led to an improvement, while impairment was predominantly seen in laboratory animals. Compared to leaves, flowers and sprouts of *Fagopyrum esculentum* Moench, grains and seeds, hull and brans and straw showed improved dietary properties. Seeds, hulls and brans of buckwheat increased the egg production rate in laying hens, as well as intake and nitrogen efficiency of broilers, and meat quality in lambs and milk quality in cows (Table 5). Whether the negative effects seen in some studies are linked to the concentration of phototoxic fagopyrins in the feeds (highest concentrations in flowers, leaves, and lowest in seeds) needs further investigation.

Less information on feed properties in rats, mice and lambs is available for *Fagopyrum tataricum*. Growth performance and feed intake were improved in lambs, but impaired in most laboratory animals.

In conclusion, *Fagopyrum esculentum* Moench and *Fagopyrum tataricum* (L.) Gaertn. exhibit a range of interesting antioxidative, antihyperlipidemic and anti-inflammatory properties and are valuable candidates for future studies in inflammatory gastrointestinal and metabolic diseases in livestock.

*Fagopyrum esculentum* may be beneficial as a feed with positive effects on the animals’ constitution (less ruminal ammonia formation, improved provision with functional fatty acids, antioxidative potential), the environment (less emissions of nitrogen and methane), and human health (beneficial fatty acid profiles of the product). Taken together, feeding of farm animals with suitable buckwheat products may contribute to a one-health approach from the nutritional side.

#### 3.5.2 Polygonum aviculare L.

Experiments with *Polygonum aviculare* L. were using the aerial parts (leaves, flowers, herbs, stem, whole plant) and roots. *In vitro* antibacterial and antifungal activities, and *in vitro* and *in vivo* anti-inflammatory properties have been reported for this species (Table 4). *In vitro* antibacterial activity against gram positive and gram negative bacteria (Additional file 3), including *E.coli* and some *Salmonella* species, *P. aviculare* might be useful in the treatment of young livestock diseases (Ayrle et al., 2016). However, the impact of *P. aviculare* on the composition of the rumen microflora remains to be studied. The plant showed *in vitro* antifungal against various *Aspergillus* sp. (Additional file 3). Secondary metabolites contained in the plants, such as tannins and flavonoid, are known to possess antibacterial and antifungal properties (Salama & Marraiki, 2010). Extracts exhibited cyclooxygenases (Tunón et al., 1995) and lowered the concentrations of pro-inflammatory cytokines (Park et al., 2018). Wound healing properties were recently reported (Seo et al., 2016), which corroborates the ethnoveterinary use in skin diseases (Mayer et al., 2014). On the other hand, P. aviculare showed no antiparasitic activity against oocysts of *Eimeria tenella* in broilers (Youn & Noh, 2001). No toxic properties of *Polygonum aviculare* L. were reported.

*Polygonum aviculare* L. lowered ammonia and methane production in rumen of sheep (Table 5). This may be due to the high concentration of polyphenols which tend to interfere with enteric methane production and proteolysis in the rumen (Macheboeuf et al., 2014; Niderkorn & Macheboeuf, 2014; ). *Polygonum aviculare* L. seems to be highly fermentable with a low methane production and may be nutritionally and environmentally beneficial when consumed by ruminants. Less information is available about feed properties in broilers and mice. For the investigated livestock, the growth performance, feed intake, and feed conversion rate was not affected, but there were positive and negative effects on growth performance of laboratory animals.

To conclude, *Polygonum aviculare* L. shows interesting antibacterial, antifungal and anti-inflammatory activities. Further studies are therefore warranted for the potential treatment of microbial infections and inflammatory gastrointestinal diseases in livestock. The reduction of methane and ammonia levels in sheep is also of interest in a health and environment perspective. There is no information about effects on livestock product quality.

#### 3.5.3 Polygonum bistorta L.

In experiments with *Polygonum bistorta* L. the aerial parts (fruits, herb), roots and rhizomes were used. *In vitro* and *in vivo* experiments showed anti-inflammatory and antidiarrheal activities (Table 4). *Polygonum bistorta* L. contains tannins and other phenolic compounds. The polyphenols may contribute to the anti-inflammatory properties (Khushtar et al., 2018) and antidiarrheal activity (Yu et al., 2005). *Polygonum bistorta* L. reportedly has antiparasitic activity against coccidial oocysts of *Eimeria spp*. in broilers (Khan et al., 2008).

Some *in vivo* investigations were conducted in the context of gastrointestinal diseases. Antidiarrheal activity in mice and on the colonic mucosa of rats was reported (Ali et al., 2015; Yu et al., 2015). Interestingly, the beneficial effect on experimental gastric ulcers in rats (Khushtar et al., 2018) supports the ethnoveterinary use of *P. bistorta* (Delafond, 1853; Mayer et al., 2014; Röll, 1866). No toxicological findings have been reported for *Polygonum bistorta* L.

The aerial parts of *Polygonum bistorta* L. show an *in vitro* ammonia and methane-lowering effect in rumen of sheep (Table 5). This may be due again to the high concentrations of polyphenols which tend to interfere with enteric methane production and proteolysis in the rumen (Macheboeuf et al., 2014; Niderkorn & Macheboeuf, 2014). *Polygonum bistorta* L. was shown to improve growth performance, but without effect on feed conversion rate in broilers.

*In summary, Polygonum bistorta* L. shows interesting anti-inflammatory and antidiarrheal activities and may thus be useful in the treatment of inflammatory gastrointestinal diseases. A lower ruminal methane and ammonia formation implies both potential benefits for animal health and the environment. *Polygonum bistorta* L. is abundant in alpine pastures and readily consumed by cattle, leading to improved fatty acid profiles in milk from grazing cows (Gorlier at al., 2012).

#### 3.5.4 Rumex obtusifolius L.

Experiments with *Rumex obtusifolius* L. were done with herbs, leaves, seeds, whole plant and roots. I*n vitro* data showed antibacterial activities (Table 4) against gram negative and gram positive bacteria (Additional file 3). Saponins and other isoprenoids, tannins, flavonoids and coumarins have been reported for the species. The high concentration of saponins and polyphenols may be responsible for the antibacterial activity (Ginovyan et al., 2020; Ginovyan & Trchounian, 2019). Due to its *in vitro* antibacterial against *E. coli, R. obtusifolius* might be interesting in the treatment neonatal diarrhea in piglets and calves (Ayrle et al., 2016). *Rumex obtusifolius* L. is an undesirable weed in fields, but has been frequently reported in ethnoveterinary and historical sources to be used to treat disorders of the gastrointestinal tract, skin, genitourinary system, udder, and the musculoskeletal and respiratory systems (Mayer et al., 2014; Röll, 1866). However, no *in vivo* data are available that would support these traditional uses. No toxicological activities have been reported for *Rumex obtusifolius* L.

*Rumex obtusifolius* L. lowered methane production in the rumen of steers (Table 5). The high content in condensed tannins is known to reduce methanogenesis and thus may have contributed to the lower methane output (Purcell et al., 2012). Also, the reduced methane output could be due to the fact that the plant does not need to be fully digested before leaving the rumen. A short ruminating time may reduce methane formation (Wilman et al., 1997).

To conclude, *Rumex obtusifolius* L. shows interesting antibacterial activities and may be useful in the treatment of infectious diseases. Little information about feed properties is available to date, but the prevention of bloating in ruminants (Waghorn & Jones, 1989) is a relevant factor in cattle nutrition and health. The methane-lowering effect may be environmentally beneficial.

#### 3.5.5 Rumex acetosa L. and Rumex acetosella L.

In experiments with these two *Rumex* species the aerial parts (herbs, leaves, whole plants) and roots were investigated. *Rumex acetosa* L. and *R. acetosella* both showed *in vitro* antibacterial activity against gram negative and gram positive bacteria, and antifungal properties (Additional file 3). In addition, *Rumex acetosa* L. showed *in vitro* and *in vivo* anti-inflammatory activity (Table 4).

Both *Rumex* species contain saponins, tannins, flavonoids, anthranoids and stilbenes. The antibacterial activity could be due, at least in part, to the polyphenols and saponins. Calves may be infected with *Salmonella* within hours after birth, which is a common cause of neonatal morbidity and mortality (Mohler et al., 2009). As *Rumex acetosella* L. is *in vitro* antibacterial against *S. enteritidis*, it might be considered as a supporting treatment option. Emodin may display anti-ulcerogenic and anti-inflammatory activities (Bae et al., 2012). These findings fit well with the ethnoveterinary use of *Rumex acetosa* L. in gastrointestinal diseases (Delafond, 1853; Mayer et al., 2014). However, investigations on beneficial effects of *R. acetosa* in parasitic and respiratory diseases are lacking, as well as studies with *Rumex acetosella* in the context of gastrointestinal diseases. For both Rumex species the influence on growth performance was investigated in rats. Administration of *Rumex acetosella* showed positive effects, whereas the effects with *Rumex acetosa* was inconsistent. No toxicological findings have been reported for both species.

To conclude, *R. acetosa* and *R. acetosella* possess potentially beneficial properties for livestock, such as antibacterial, antifungal and anti-inflammatory activities. No information is currently available about livestock performance, product quality and ruminal biotransformation.

## 4 Conclusion

In the European alpine regions, some 50 species of the Polygonaceae family occur, most of them wild and very few cultivated. The spectrum of phytochemicals in the family renders these plants interesting as options for the treatment and nutrition of farm animals. Our qualitative systematic review showed that the majority of published data was dealing with pharmacological activities, while fewer publications were addressing specific veterinary issues related to animal nutrition and disesae prevention. For two cultivated (*Fagopyrum spp.*) and five wild (two *Polygonum spp.* and three *Rumex spp.*) species desirable effects for veterinary treatment and animal nutrition were reported. Beneficial effects of plants may be multifacetted. For example, antibacterial properties may be beneficial in the prevention of gastrointestinal infection in young animals on the one hand, and increase nutrient conversion in ruminants on the other. Possible, but not yet evaluated preventive properties against gastrointestinal bacterial infections in piglets (*Fagopyrum spp.*) or parasite infections in chicken (*Polygonum bistorta*) could be provided by the plants when used as a feed or feed additive. In ruminants, proven modulating effects on the digestive process by phytochemicals from Polygonaceae may positively impact animal health and resilience, the environment and climate, as well as the health of human consumers of the animal products. This perspective on promising yet under-utilized plants as feed and preventive treatment implies that a One Health approach can be developed and applied also at the level of agricultural animal systems.

## Ethical statement

Due to the fact that the submitted manuscript is a review article, an ethical statement seems to be not applicable for the authors.

## Additional files

**Additional file 1:** Protocol of systematic review

**Additional file 2:***In vivo* experiments

**Additional file 3:***In vitro* experiments

## CRediT authorship contribution statement

**Zafide Türk:** Writing – original draft, Visualization, Investigation, Formal analysis, Data curation. **Florian Leiber:** Writing – review & editing, Supervision, Methodology, Funding acquisition, Conceptualization. **Theresa Schlittenlacher:** Writing – review & editing, Visualization, Resources, Project administration. **Matthias Hamburger:** Writing – review & editing, Supervision, Methodology, Conceptualization. **Michael Walkenhorst:** Writing – review & editing, Writing – original draft, Supervision, Project administration, Data curation, Conceptualization.

## Declaration of competing interest

The authors declare to have no conflict of interest.

## Appendix Supplementary materials

Image, application 1

Image, application 2

Image, application 3
