# Supplementary material for: Multiple benefits of herbs: Polygonaceae species in veterinary pharmacology and livestock nutrition
Source: Vet Anim Sci. 2024 Dec 2;27:100416. doi: 10.1016/j.vas.2024.100416 (PMC11667078; doi:10.1016/j.vas.2024.100416)
Supplement: Supplementary file 1 [file mmc1.pdf]

Protocol of the systematic review

Aim of the systematic review

The aim of this systematic review is to estimate based on recent *in vitro*, *in vivo* and clinical research, how far Swiss native or cultivated Polygonaceae species might be promising candidates (a) to elevate feed diversity and richness in secondary metabolites, (b) improve livestock performance and quality of livestock products and (c) to treat and prevent livestock diseases.

PICOS SCHEME

(Created with Michael Walkenhorst and Florian Leiber on 14.01.2021)

Population
Ruminants, livestock, cow, cattle, heifer, calf, pig, sow, piglet, poultry, laying hen, broiler, sheep, goat, lamb, rabbit

Intervention
plant material from Swiss Polygonaceae species and extracts based on this plant material

Comparator
Standard, placebo, untreated

Outcomes
Effects, potentially related with livestock health and performance.

Study design
In vitro (including rumen model), phytochemical, *in vivo*, clinical studies

Procedure

1. **Which are the relevant plant species?**
   1.1. **Inclusion of all Polygonaceae species which belong to the Swiss normal flora (Infoflora.ch)**

   1.2. **Screening of relevant further sources based on primary and secondary literature with regard to further Polygonaceae species**
   - Three historic veterinary pharmacology textbooks
   - Two publications focussing on European ethnoveterinary plant use
   - One publication based on alpine forage plant species
   - A list of all used plants, based on recent Swiss ethnoveterinary research

   1.2.1. **Collecting data about plants within these sources concerning**
   - Plant species
   - Plant part
   - Indication, classified according to the ATC vet Code system (QA, QR, QL etc.)
   - Administration

   1.3. **Screening of seed mixtures for feed producing grassland and wildflower (UFA Semen) with regard to further Polygonaceae species**

   ⇨ Finally we selected 51 Swiss Polygonaceae species.

2. Searching for publications in online databases

Scientific information about these Polygonaceae species were searched in the PubMed and the Web of science databases.

The search was conducted in the time between 20.01.2021 until 27.01.2021:

3.1 PubMed

3.1.1 Search with a search term

("Latin name" OR "English name" OR "herbal drug") NOT (cancer* OR anticancer* OR anti-cancer* OR anti-tumor* OR antitumor* OR tumor*)

English name and herbal drug = if available

Searches for knotweed, knotgrass and sorrel were conducted separately with this search term: ("knotweed" OR "knotgrass" OR "sorrel") NOT (cancer* OR anticancer* OR anti-cancer* OR anti-tumor* OR antitumor* OR tumor*).

3.2 Web of Science od Web of Knowledge

3.2.1 Search with a search term

("Latin name" OR "English name" OR "herbal drug") NOT (cancer* OR anticancer* OR anti-cancer* OR anti-tumor* OR antitumor* OR tumor*)

English name and herbal drug = if available

Searches for knotweed, knotgrass and sorrel were conducted separately with this search term: ("knotweed" OR "knotgrass" OR "sorrel") NOT (cancer* OR anticancer* OR anti-cancer* OR anti-tumor* OR antitumor* OR tumor*).

The following research areas were included in the search within Web of Science:

Agricultural, Agriculture diary animal science, Agriculture multidisciplinary, Agronomy, Allergy, Behavioral science, , Biochemical research methods, Biochemistry molecular biology, Biology, Biotechnology applied microbiology, Cell biology, Chemistry analytical, Chemistry applied, Chemistry medicinal, Chemistry multidisciplinary, Chemistry organic, Chemistry physical, Dermatology, Engineering, Engineering chemical, Entomology, Food science technology, Green sustainable science technology, Immunology, , Integrative complementary medicine, Microbiology, Multidisciplinary science, Mycology, Nutrition dietetics, Pharmacology pharmacy, Physiology, Substance abuse, Surgery, Toxicology, Veterinary science, Virology, Zoology

All detected publications were saved in an EndNote 20 database. A group was created for each of the Polygonaceae species, knotweed, knotgrass and sorrel.

3. Removing of duplicates with help of EndNote

Within the Polygonaceae groups, the duplicates were removed automatically with EndNote. Not all duplicates could be removed automatically, so they were removed manually afterwards. Before the automatical process of removal of duplicates, the folders knotweed, knotgrass and sorrel were assigned to the corresponding folders of Polygonaceae species, if the species could be clearly identified.

4. Definition of inclusion and exclusion criteria by three person

Overall only peer-reviewed publications, which are not reviews and which offer an abstract, written in English, German, Turkish, French or Spanish (if available) language were considered for the further evaluation. In- and exclusion criteria were pre-defined by three scientists. If the publications do not fit those criteria, they are excluded manually: First step screening of the title ad second step screening of the abstract, some further publications were excluded during the full-paper evaluation. The amount of publications remaining after the first step and after the second step are sampled.

4.1. Inclusion of publications which:

- Provide an abstract written in English
- Are published in peer-reviewed journals
- Investigate at least one of the Plant species (Swiss Polygonaceae), as raw material or in form of preparations like extracts or one single component

- Report about trials which compare a Polygonaceae species based treatment with a control treatment (placebo, no or standard treatment)

  And dealing with:

  a)
  - *in vitro* (including rumen model), *ex vivo*, *in vivo*, clinical trials

  AND
  - Antimicrobial/antibacterial/antifungal/antiparasitic effects with regard of animal and human pathogens
  - Anti-inflammatory effects
  - Astringent effects
  - Anti-oxidative effects
  - Immunomodulating effects
  - Effects in the gastrointestinal tract
  - Effects in the respiratory tract
  - Dermatological effects
  - Hepatoprotective effects
  - Influence of methane or CH4, Ammonia, Ammonium, NH3, NH4, Polyunsaturated fatty acid or PUFA, conjugated linoleic acid or CLA in rumen models or in livestock
  - Influence of ruminal biohydrogenation
  - Influence on fermentation in ruminants (rumen, ruminant, rumen and protein)
  - Over all animal nutritional effects
  - Effects on ruminal physiology
  - Effects on over all animals physiology
  - Overall effects on animal or human physiology
  - Effects on livestock product (meat, milk, egg quality)
  - repellent, insecticide or anti-parasitic effects
  - Plants used as food

  OR

  b)
  - Ingredients, constituent, components of plants and the detection or extraction of them
  - Toxic activity or side effects

4.2. Excluding criteria

Exclusion of all publications which:

- Do not provide an abstract
- Are not published in peer-reviewed journals
- Investigate a mixture of different plants species in a combined preparation
- Dealing with other plant species or subspecies than the focused
- Do not have a control group
- Do not mention a name clearly distinguishing the species or the Latin or drug name of the plant (e.g. only mentioning “Knotweed” and not “Alpine knotweed”)

Or dealing with:

- Plants genetic
- Cultivation or breeding of plants
- Weed
- Plant pathology, fertilizer, plant protection systems or pesticides
- Ecology
- Geology
- Ethology
- Sociology
- Ethnobotany

- Food quality, technology or food packaging
- Homeopathic use of the plants (excluding mother-tinctures, papers on mother tinctures are included)
- Cancer, tumor, anticancer effects, antitumor effects
- Vasorelaxing, anti-atherosclerotic, anti-fibrotic effects
- Technical and analytical methods
- Allergy, pollen
- Ophthalmic, human specific antiviral effects, anti-influenza effects, blood products and properties
- Heavy metals
- Soil science
- Radical scavenging effects

5. Exclusion of publications with manual title and abstract check within EndNote

The predefined inclusion and exclusion criteria were used for manual title check. To structure the title check within each individual plant species folder, search words that occurred repeatedly in titles or abstracts of publications were used to bundle a couple of similar publications with regard to one topic, which were screened in one working step afterwards. This process was conducted regularly with one supervisor.
For example: During the reading of 10 titles, it was noticed that the word "weed" occurs frequently and deals with weed control, which is not of our interest. With the search word "weed" a bundle of 100 publications is formed, which are manually checked for interesting and uninteresting publications afterwards (77 of 100 publications were excluded, because they did not match the inclusion criteria).

<u>The following search words were used to find thematically related publications within each plant species folder which were afterwards checked manually in one block. During this process some publications were excluded while others were included for the final analysis:</u>

<u>28.01.2021: *Polygonum aviculare* L.:</u>
"weed" not "knotweed": 77 publications were excluded
"Seed": 20 publications were excluded
"metal": 5 publications were excluded
"herbicide": 4 publications were excluded

73 publications were excluded without building thematically related blocks

<u>29.01.2021: *Rumex conglomeratus* MURRAY:</u>
"Screening": 9 publications were excluded
"dock": 29 publications were excluded
"structure" or "structural": 13 publications were excluded
"binding": 8 publications were excluded
"gene" or "genomic": 5 publications were excluded
"cluster": 8 publications were excluded
"interaction": 5 publications were excluded

34 publications were excluded without building thematically related blocks

<u>29.01.2021: *Reynoutria japonica* Houtt.:</u>
"invasive" or "invasion": 31 publications were excluded
"biological control": 8 publications were excluded
"gene" or "genomic": 4 publications were excluded
"effect": 6 publications were excluded
"cuspidatum": 8 publications were excluded
"giant knotweed" or Fallopia sachalinensis": 4 publications were excluded

73 publications were excluded without building thematically related blocks

<u>29.01.2021: *Polygonum bistorta* L.:</u>
"weed" not "knotweed": 68 publications were excluded

“gutierrezia”: 5 publications were excluded
“soil”: 4 publication were excluded

51 publications were excluded without building thematically related blocks

<u>01.02.2021: Rumex sp.:</u>
“weed”: 7 publications were excluded
“extract”: 5 publication were excluded
“Hibiscus”: 11 publications were excluded
“pollen”: 10 publications were excluded
“gene” or “genomic: 5 publications were excluded
“Oxalis”: 8 publications were excluded
“effect”: 6 publication were excluded

98 publications were excluded without building thematically related blocks

<u>01.02.2021: *Polygonum minus* Huds.:</u>
“weed”: 46 publications were excluded
“control”: 5 publications were excluded
“herbicide”: 5 publications were excluded
“anti”: 9 publication were excluded
“chemical”: 6 publications were excluded
“extract”: 8 publications were excluded
“gene” or genomics: 8 publications were excluded

64 publications were excluded without building thematically related blocks

<u>01.02.2021: *Polygonum hydropiper* L.:</u>
“anti”: 14 publications were excluded
“effect”: 8 publications were excluded
“activity”: 5 publications were excluded
“method”: 3 publications were excluded
“accumulation”: 8 publication were excluded

90 publications were excluded without building thematically related blocks

<u>02.02.2021: *Rumex cripus* L.:</u>
“root”: 12 publications were excluded
“soil”: 7 publications were excluded
“effect”: 14 publications were excluded
“control”: 7 publications were excluded
“weed”: 16 publications were excluded
“extract”: 11 publications were excluded
“germination”: 5 publications were excluded
“seed”: 11 publications were excluded

80 publications were excluded without building thematically related blocks

<u>02.02.2021: R*umex acetosa* L.:</u>
“metal”: 11 publications were excluded
“chromosome”: 29 publications were excluded
“gene” or “genom”: 13 publications were excluded
“DNA” or “RNA”: 6 publications were excluded
“effect”: 15 publications were excluded
“growth”: 9 publications were excluded
“species”: 8 publications were excluded
“grass”: 7 publications were excluded

77 publications were excluded without building thematically related blocks

03.02.2021: *Rumex obstusifolius* L.:
"root": 9 publications were excluded
"effect": 43 publications were excluded
"control": 20 publications were excluded
"seed": 18 publications were excluded
"weed": 14 publications were excluded
"beetle": 6 publications were excluded
"virus": 4 publications were excluded
"leaf": 5 publications were excluded

77 publications were excluded without building thematically related blocks

03.02.2021: *Fallopia dumetorum* (L.) Holub:
"weed" not "bindweed": 49 publications were excluded
"control": 53 publications were excluded
"herbicide": 15 publications were excluded
"effect": 22 publications were excluded
"convolvulus": 37 publications were excluded
"field": 22 publications were excluded
"candidatus": 16 publications were excluded
"bois noir" or "disease" or "infection": 12 publications were excluded
"phytoplasma": 7 publications were excluded

78 publications were excluded without building thematically related blocks

05.02.2021: *Rumex acetosella* L.:
"weed": 17 publications were excluded
"soil": 7 publications were excluded
"effect": 7 publications were excluded

55 publications were excluded without building thematically related blocks

05.02.2021: *Rumex palustris* Sm.:
"gene": 28 publications were excluded
"bio": 8 publications were excluded
"ethylene": 15 publications were excluded
"flooding": 12 publications were excluded

29 publications were excluded without building thematically related blocks

05.02.2021: *Polygala senega* L.:
"neuro": 13 publications were excluded
"study" or "studies": 8 publications were excluded

61 publications were excluded without building thematically related blocks

05.02.2021: *Polygonum persicaria* L.:
"weed": 29 publications were excluded
"seed": 5 publications were excluded
"phenotypic": 7 publications were excluded
"gene": 5 publications were excluded

36 publications were excluded without building thematically related blocks

05.02.2021: *Reynoutria sachalinensis* (F.Schmidt) Nakai:
"weed": 7 publications were excluded

“soil”: 5 publications were excluded
“gene” or “genotype”: 5 publications were excluded
“powdery mildew”: 14 publications were excluded

35 Publications were excluded without building thematically related blocks

08.02.2021: *Polygonum orientale* L.:
“control”: 5 publications were excluded
“adsorption”: 5 publications were excluded
“determination”: 5 publications were excluded

41 Publications were excluded without building thematically related blocks

08.02.2021: *Polygonum perfoliatum* L.:
“weed”: 17 publications were excluded
“control”: 5 publications were excluded

30 Publications were excluded without building thematically related blocks

08.02.2021: *Rumex patientia* L.:
“root”: 6 publications were excluded
“soil”: 6 publications were excluded

30 Publications were excluded without building thematically related blocks

08.02.2021: *Polygonum lapathifolium* L.:
“weed”: 20 publications were excluded

23 Publications were excluded without building thematically related blocks

09.02.2021: *Fagopyrum tataricum* (L.) Gaertn.:
“seed”: 37 publications were excluded
“gene” or “genom”: 74 publications were excluded
“effect”: 36 publications were excluded
“anti”: 13 publications were excluded
“breeding”: 6 publications were excluded
“determination”: 8 publications were excluded
“root”: 9 publications were excluded
“bio”: 12 publications were excluded
“grain”: 10 publications were excluded
“DNA” or “RNA”: 9 publications were excluded

65 Publications were excluded without building thematically related blocks

09.02.2021: *Fallopia convolvulus* (L.) A. Löve:
“weed” or “bindweed”: 91 publications were excluded
“herbicide”: 27 publications were excluded
“control”: 41 publications were excluded
“convolvulus arvensis”: 34 publications were excluded
“gene”: 12 publications were excluded
“glyphosate”: 17 publications were excluded
“bio”:10 publications were excluded
“candidatus” or “bois noir”: 24 publications were excluded
“growth”: 6 publications were excluded
“seed”: 6 publications were excluded
“effect”: 10 publications were excluded
“phyto”: 9 publications were excluded
“field”: 12 publications were excluded

61 Publications were excluded without building thematically related blocks

10.02.2021: *Fagopyrum esculentum* Moench:
“gene” or “genom”: 256 publications were excluded
“method”: 103 publications were excluded
“process”: 102 publications were excluded
“seed”: 331 were excluded

11.02.2021: *Fagopyrum esculentum* Moench:
“tataricum” or “tartary”: 344 publications were excluded
“allerg” or “IgE” or “Ig-E” or “anaphylax”: 199 publications were excluded
“effect”: 386 publications were excluded

14.02.2021-18.02.2021
“Buckwheat” in title and abstract: 2139 publications were included, 262 publications were excluded

⇨ Folders were created in EndNote 20 with:
Publications (livestock)
Publications (*in vivo* trial)
Publications (*in vitro* trial)
Publications (Trial with human and human food)
Publications (primary and secondary metabolites)
Publications (Food)

1325 publications were rearranged in these groups and manual title screening was conducted. 814 publications could not be allocated.

19.02.2021: Within folder with 814 publications:
“weed”: 40 publications were excluded
“food”: 86 publications were excluded
“honey”: 47 publications were excluded
“control”: 79 publications were excluded
“soil”: 56 publications were excluded
“cultiv”: 36 publications were exclude
“detect”: 30 publication were exclude
“root”: 18 publications were excluded
“typ”: 35 publications were excluded
“mix”: 17 publications were excluded
“anti”: 27 publications were excluded
“pollen”: 10 publications were excluded

Total: 1266 publications were excluded without building thematically related blocks

22.02.2021: *Polygonum rurivagum* Boreau:
“weed”: 64 publications were excluded
“gene” OR “genom”: 205 publications were excluded
“root”: 109 publications were excluded
“Polygonum”: 729 publications were excluded
“seed”: 38 publications were excluded
“anti”: 93 publications were excluded
“effect”: 75 publications were excluded
“Polygoni”: 64 publications were excluded
“extract”: 76 publications were excluded

28 publications were excluded without building thematically related blocks

22.02.2021: *Rumex nebroides* Campd.:

“weed”: 56 publications were excluded
“gene” OR “genom”: 184 publications were excluded
“root”: 101 publications were excluded
“seed”: 42 publications were excluded
“control”: 41 publications were excluded
“Rumex”: 121 publications were excluded
“anti”: 53 publications were excluded
“eefect”: 27 publications were excluded

94 publications were excluded without building thematically related blocks

23.02.2021: *Rheum officinale/palmatum* L.:
“gene” OR “genom”: 109
“growth”: 54 publications were excluded
“method”: 139
“extract”: 66

182 publications were excluded without building thematically related blocks

Abstract check:
All reviews (n= 41), publications solely about side effects/toxicology (n= 75), food processing (n=223), pharmacognostics (n= 212) and other publications (n= 153) which did not match the defined criteria were excluded.

6. Data processing

Trials investigating effects of preparations from Polygonaceae species on diseases or other measurement parameters in living animals and humans were categorized as “*in vivo*”. Investigations using pathogens, cell layer or *ex vivo* models were categorized as “*in vitro*”. Subgroups were formed for *in vivo* (including (a) livestock, (b) laboratory animals and (c) human) and *in vitro* (including (a) livestock directed research like rumen model or livestock cell lines, (b) laboratory animal cell lines, and (c) human cell lines and (d) further research mainly based on bacterial, fungal, viral culture).

The finally included publications were categorized by one person into two main groups: “*in vivo*” and “*in vitro*” publications. *In vivo* publications were publications with *in vivo* data even if they included in addition *in vitro* data, *in vitro* publications describing solely *in vitro* data. As there are publications that describe several trials and not seldom both *in vitro* and *in vivo* data, the main research unit of our review is the “experiment” as defined as follow:

In vivo experiment = Each trial within the same publication documenting two or more groups of animals kept under the same conditions during the same time period, receiving raw material or an extract of one plant part (or the whole plant) of a Polygonaceae species (publication x trial x plant species x plant part x extracting agent) compared to a control group.

In vitro experiment = Summary of all *in vitro* trials documented within the same publication and conducted with a specific extract of a specific plant part (or the whole plant) of one Polygonaceae species (publication x plant species x plant part x extracting agent).

All effects and their expressions (activities) were listed in a table for *in vivo* experiments (Additional file 2)

- Per each experiment all available information were systematically gathered with particular regard to general effects and effects potentially relevant for livestock.
- The expression of the effects were divided into four different levels: Plus (+) means there is a required effect, zero (0) means there is no effect observed, minus (-) means there is the opposite

of the required activity observed and question mark (?) means there is an inconsistent activity (Table 2).

General effects:
- Antioxidative
- antiviral
- antibacterial (pathogenes)
- antifungal
- antiparasitic
- immunomodulating effects
- hepatoprotective
- anti-inflammatory
- antihyperlipidemic
- prebiotic
- probiotic
- antidiarrheic
- smooth muscle reactivity
- astringent

Livestock related effects
- meat oxidative stability
- milk production
- milk quality
- meat quality
- egg quality
- performance
- feed intake
- feed conversion rate
- physiological effect on the rumen

All effects and their expressions (activities) were listed in a table for *in vitro* experiments (Additional file 3)
- Per each experiment all available information were systematically gathered with particular regard to general effects and effects potentially relevant for livestock.
- The expression of the effects were divided into four different levels: Plus (+) means there is a required effect, zero (0) means there is no effect observed, minus (-) means there is the opposite of the required activity observed and question mark (?) means there is an inconsistent activity (Table 2).

General effects:
- antioxidative
- antiviral
- antibacterial
- antibiotic modulatory effects
- antifungal
- antiparasitic
- immunomodulating effects
- hepatoprotective
- anti-inflammatory
- antihyperlipidemic
- smooth muscle reactivity

Livestock related effects
- physiological effect on the rumen
    - Reduction of ammonia level
    - Reduction of methane level
